# Extracellular vesicle-based drug overview: research landscape, quality control and nonclinical evaluation strategies

**DOI:** 10.1038/s41392-025-02312-w

**Published:** 2025-08-14

**Authors:** Gangling Xu, Jing Jin, Zhihao Fu, Guangming Wang, Xinhua Lei, Jun Xu, Junzhi Wang

**Affiliations:** 1 National Institutes for Food and Drug Control, Beijing, China; 2 State Key Laboratory of Drug Regulatory Science, Beijing, China; 3 National-Local Joint Engineering Laboratory of Animal Models for Human Disease, The First Hospital of Jilin University, Jilin, Changchun China; 4 East Hospital, Stem Cell Research Center, School of Medicine, Tongji University, Shanghai, China

**Keywords:** Drug regulation, Mesenchymal stem cells

## Abstract

Extracellular vesicles share lipid‒protein membranes with their parent cells, allowing for the targeted transfer of bioactive cargo to recipient cells for functional modulation. The biological features allow extracellular vesicles to serve both as intrinsic therapeutics and as engineered delivery vehicles for targeted molecule transport. In recent years, extracellular vesicle-based therapy has shown great potential as a new therapeutic approach for traumatic conditions and degenerative, acute, and refractory diseases. As extracellular vesicle engineering continues to evolve, more innovative drugs are expected to receive investigational new drug approvals and marketing approvals from regulatory agencies in the future. However, many challenges exist in terms of mechanistic understanding, engineering modifications, manufacturing processes, quality control, and nonclinical research, and no drug regulatory authorities have currently issued specific technical evaluation guidelines for extracellular vesicle-based drugs, all of which have hindered the clinical translation of these drugs. In this article, which is focused primarily on extracellular vesicles derived from mammalian cells, we summarize the clinical translation and process development research status of extracellular vesicle-based drugs and propose both general considerations and key aspects of quality control strategies and nonclinical evaluations in the development process. The aim of this review is to provide valuable references for the development and evaluation of extracellular vesicle-based products, accelerate the clinical translation process, and benefit patients as soon as possible.

## Introduction

Innovative therapeutic products, such as antibody‒drug conjugates (ADCs)^1,2^, gene therapies^3,4^, stem cells^5,6^, and immune cell therapy products^7,8^, provide new options and hope for the treatment of many diseases that are otherwise difficult to treat. With the development of extracellular vesicles (EVs) as natural therapeutics or delivery tools, EV-based drug research and development has become a popular research spot^9,10^. EV drugs share properties with these innovative therapeutic products by possessing the targeted delivery characteristics of ADCs, serving as carriers for gene therapy, and inheriting the function of source cells. In addition, they have unique characteristics, such as a low risk of tumorigenicity and embolism, low immunogenicity, good biocompatibility, and rich bioactive targets^10,11^. Therefore, EV drugs have great potential as innovative therapeutic products.

EVs have a phospholipid bilayer plasma membrane structure and are released by cells but cannot replicate^12^. It is believed that EVs, which are composed of microvesicles and exosomes, are produced by all living cells. Microvesicles are formed and released after intracellular substances are directly enveloped by the cell membrane, with sizes ranging from 30 to 150 nm in diameter. Exosomes are formed by endocytosis, in which substances are enveloped by the plasma membrane to form membranous particles inside an endosome. The endosome fuses with the cell membrane and releases membrane-bound particles, which range in size from 100 to 1000 nm^13^. Microvesicles and exosomes are very similar in their structure and composition, and the heterogeneity of EVs makes their classification challenging (Table 1). Because it is almost impossible to determine whether EVs are microvesicles or exosomes, which is consistent with the latest naming rules of the International Society for Extracellular Vesicles (ISEV), all the exosomes or microvesicles involved in this research are referred to as EVs in this paper.

**Table 1:** The heterogeneity of EVs (microvesicles and exosomes) parameters

Heterogenous Parameter	Specific Features	Influencing Factors	Implications for Downstream Development
Size distribution	30–1000 nm; overlap between exosomes and microvesicles	Biogenesis pathway, isolation methods	Affects separation strategies and purity assessment
Surface markers	Shared expression of CD9, CD63, CD81 across subtypes	Cell type, phenotypic changes	Impacts identification and subpopulation profiling
Cargo composition	Uneven distribution of proteins, RNA, DNA, and lipids	Parental cell state, environmental conditions	Determines functional effects and therapeutic indications
Biological function	e.g., Pro- or anti-inflammatory effects, immune modulation, metastasis	Cargo content, recipient cell interaction	Guides clinical use and therapeutic design
Method sensitivity	Varying detection or capture efficiencies across isolation and analysis tools	Instrument sensitivity, parameter selection	Contributes to interlab variability and reproducibility issues

*EVs* extracellular vesicles

The lipid bilayer membrane structure of EVs is similar to that of their parent cells, which contain sphingolipids, cholesterol, phospholipids, and membrane proteins. EVs are rich in protein, RNA, and DNA and can target specific cells through their plasma membrane characteristics. EVs then transfer active molecules to target cells to regulate their biological functions^14,15^. These biological properties are integral to two important applications of EVs in the treatment of refractory diseases, such as degeneration, inflammation, and tumorigenesis: as a natural therapeutic drug and as a drug delivery tool^16^. As therapeutic drugs, EVs themselves can be therapeutic, and more importantly, they can be engineered to effectively load key molecules and improve drug delivery, thereby improving their therapeutic effect. As a drug delivery tool, EV-based drug engineering can enable drugs to penetrate target cells more effectively—thus exerting optimal efficacy—and reduce side effects caused by drugs delivered to nontarget cells, therefore improving the safety of treatment. At present, although natural therapeutic EV products have moved toward clinical transformation, engineered EV therapeutic drugs may have greater potential for clinical use^17–19^.

As potential therapeutic products, many companies are developing EV-based drugs. There are currently more than 100 clinical studies worldwide evaluating the use of natural and engineered EV drugs to treat respiratory diseases, nervous system diseases, severe acute inflammation, and tumors. However, these treatments face technical challenges, such as defining the molecular mechanism of action, immature large-scale production technology, and difficult engineering modifications.

In addition, there are considerable challenges related to regulatory science, including unclear product definitions and classifications and a lack of technical guidelines related to product research and development (R&D) (none of the global drug regulatory agencies have issued any EV drug guidelines), which restrict the clinical development of EV drugs to varying degrees. In this paper, we reviewed the development of EVs as therapeutic drugs or drug delivery vehicles (excluding their use in the clinical diagnosis of diseases). Furthermore, on the basis of the basic research and clinical translation of EV drugs and the progress of technology associated with their production, we propose both general and specific considerations related to their production process, quality control, and nonclinical research supporting their development, with the aim of promoting EV drug development.

## Progress in the research and development of EV drugs

**Fig. 1:**
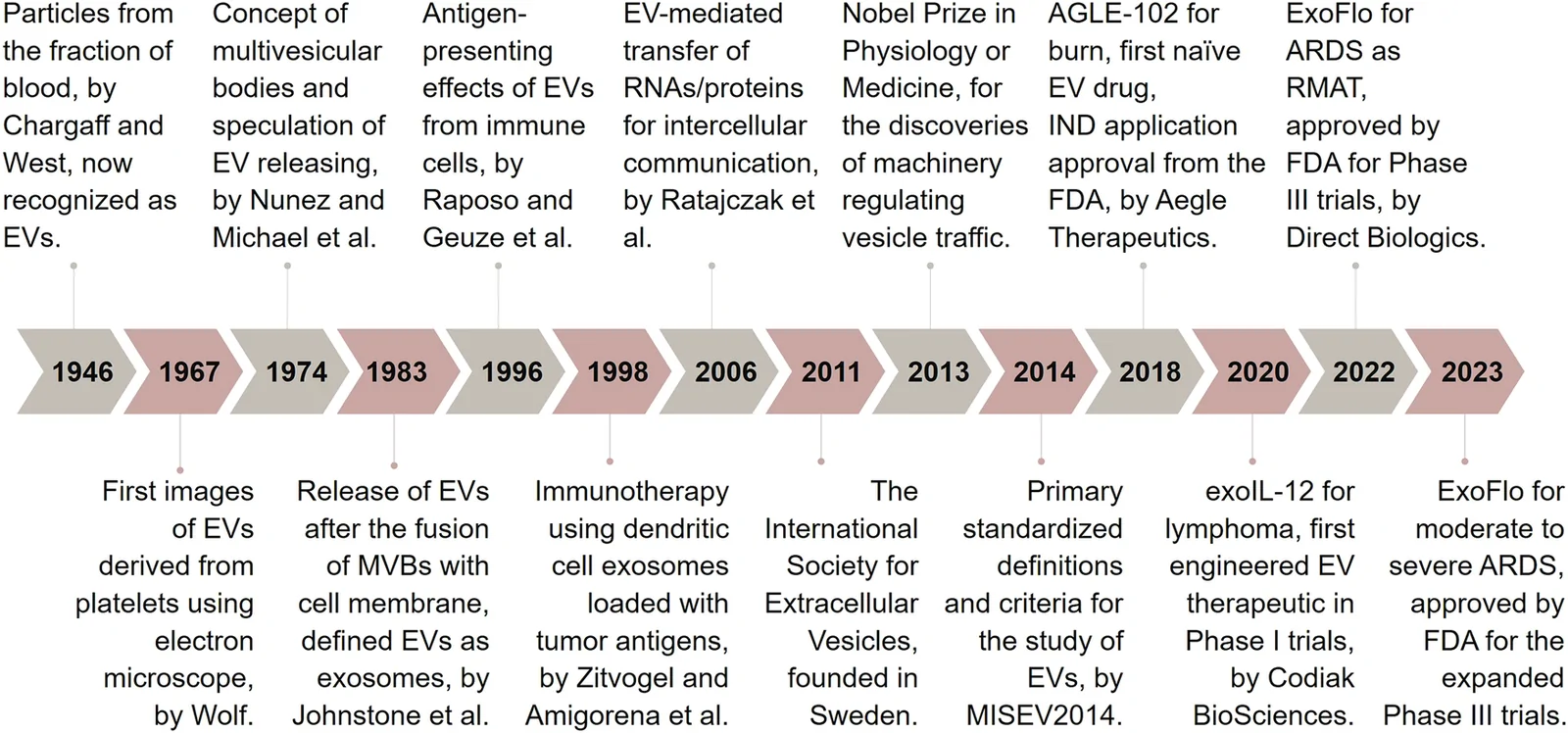
Key milestones in the development of extracellular vesicle (EV)-based drugs. The figure displays a chronological timeline highlighting significant milestones or events in EV drug development from 1946 to 2023. Each marked year represents a pivotal development. (IND investigational new drug, ARDS acute respiratory distress syndrome, RMAT regenerative medicine advanced therapy)

EVs were first discovered in the 1950s, and for decades, they were initially considered “garbage bins” of cell metabolites^20,21^. In the late 1990s, researchers discovered that EV membranes were embedded with transmembrane proteins and rich in proteins, lipids, and nucleic acids, which led to the gradual discovery of related physiological functions^22–26^. The Nobel Prize in Physiology or Medicine was awarded to James E. Rothman, Randy W. Schekman, and Thomas C. Südh of for their contributions to the discovery and elucidation of EV transport and regulatory mechanisms, which reintroduced EVs to the world as a type of “courier” that freely shuttled between cells. The discovery of EVs and the redefinition of their functions changed our understanding of cell-to-cell communication and opened new frontiers in cell biology, molecular biology, and medical research^27,28^. From the initial “metabolic waste bins” to the currently advanced drugs, EV research and development has undergone a long period of exploration (Fig. 1). Here, we first review the development of EV drugs, including their progression from natural to engineered EVs and the transition from basic research to drug development.

### Natural EVs

Theoretically, all cells of an organism secrete natural EVs. Under physiological conditions in vivo, natural EVs released by specific types of cells can be absorbed by neighboring cells directly or by distant cells through fluid circulation to perform their biological functions in target cells^29^. Studies have sought to reveal the mechanism and effects by which natural EVs regulate the microenvironment in vivo. For example, natural EVs released by tumor cells in vivo were shown to mediate immunosuppression, immune escape, and drug resistance by regulating the tumor microenvironment^30,31^. It is difficult to translate natural EVs in such studies to disease treatment. Therefore, the natural EVs discussed in this paper refer mainly to EVs isolated and purified from cell culture solutions, biological fluid, or plant tissue lysates without further modifications. Various cell cultures (mammalian cells, insect cells, microbial cells), biological fluids (blood, urine, saliva, cerebrospinal fluid, milk), and plant tissues (fruits, leaves, rhizomes) are currently used to prepare EVs^32^. Here, we discuss the main natural EV species used for disease treatment research, including mammalian cell-derived EVs, milk-derived EVs, and plant-derived EVs, and consider the prospects of their large-scale production and clinical applications (Table 2).

**Table 2:** Classification and summary of natural EVs used for therapeutic applications

EVs Type	Source	Key Biological Functions	Therapeutic Applications	Advantages	Limitations
Mammalian cell (MSC-EVs)	Human mesenchymal stem cells(e.g., adipose, bone marrow, umbilical cord)	Anti-inflammation, immunomodulation, tissue repair	Injury repair (lung, liver, kidney, central nervous system, skin), inflammatory- and aging-related diseases	Broad sources, multiple functions, wide clinical applicability, close to achieving industrial-level production	High production cost, upstream culture required
Mammalian cell (NSC-EVs)	Human neural stem cells (fetal tissue or induced pluripotent stem cells)	Neuroprotection, neuroregeneration, anti-neuroinflammation	Stroke, spinal cord injury, Alzheimer’s, Parkinson’s	Rich in neural-specific miRNAs, potential neurological targeting	Ethical source concerns, low scalability
Mammalian cell (EPC-EVs)	Human endothelial progenitor cells (peripheral and umbilical blood)	Pro-angiogenesis, anti-inflammation, anti-apoptosis	Ischemic diseases, myocardial infarction, diabetic complications	Express angiogenesis-related factors, vascular targeting	Limited cell availability, immature large-scale culture
Mammalian cell (Immune cell-derived EVs)	Dendritic cells, T cells, NK cells	Immune activation or suppression, anti-tumor	Cancer immunotherapy	Enhance anti-tumor immunity via various pathways	Low yield, immune safety concerns
Milk-derived EVs	Human or bovine milk	Anti-inflammation, antioxidant, anti-fibrosis, anti-tumor, tissue repair	IBD, liver and cardiac fibrosis, skin and bone repair, cancer	Abundant, oral delivery, bioactive content, high stability	Need for large-scale purification, animal-derived product control
Plant-derived EVs	Edible plant tissue (e.g., ginger, orange, cabbage)	Anti-tumor, anti-inflammation, antioxidant, regeneration	Liver and skin injuries, gastrointestinal inflammation, drug delivery	Inexpensive, rich in phytochemicals, low immunogenicity, scalable	Complex purification, unproven targeting, limited engineering capability

*EVs* extracellular vesicles, *MSC-EVs* mesenchymal stem cell-derived extracellular vesicles, *NSC-EVs* Neural stem cell-derived EVs, *EPC-EVs* Endothelial progenitor cell-derived EVs

#### Mammalian cell**-**derived EVs

In this review, EVs derived from mammalian cells refer to EVs isolated and purified from mammalian cell culture supernatants in vitro. EVs derived from mammalian cells are the most widely used and well-studied type of EV. A variety of mammalian cells, including human and various mammalian animal models, are commonly used to produce EVs for research. Here, to focus on eventual clinical applications, we provide a research overview of EVs derived from human cells.

##### Mesenchymal stem cell (MSC)-derived EVs

MSCs are mesenchymal cells with multilineage differentiation potential and excellent immune regulation ability, which creates an appropriate microenvironment for tissue repair and regeneration^33^. MSCs are widely used in cell therapy and can be isolated and cultured from a range of sources, including human fat, bone marrow, the umbilical cord, placenta, dental pulp, and other tissues. Researchers have gradually come to believe that the role of MSCs in treating various diseases may be mediated primarily by EVs through paracrine signaling^34,35^. Recent studies have shown that MSC-derived extracellular vesicles (MSC-EVs) have the same therapeutic effects as their parent cells do, including anti-inflammatory, immunomodulatory, and tissue repair effects, which are manifested by a variety of functions, including inhibiting the polarization of proinflammatory macrophages and promoting the polarization of anti-inflammatory macrophages, regulating the proliferation and differentiation of helper T cells, promoting the proliferation of target cells, mediating antiapoptotic effects, and promoting epithelial‒mesenchymal transition and angiogenesis^36^. Consequently, human MSC-EVs have been directly applied in basic research and clinical treatment of various tissue and organ injuries, along with inflammatory and aging-related diseases, such as those of the lung^37–39^, liver^40–42^, kidney^43–45^, bone^46–48^, skin^49–52^, heart^53–56^, ovary^57–59^, and central nervous system^36,60–62^. MSC-EVs are among the most important types of natural EV drugs and have relatively high production capacity, diverse functions, and broad clinical applications. MSC-EV-based EV drugs are closest to achieving clinical translation and industrial production.

##### Neural stem cell-derived EVs

Neural stem cells (NSCs) differentiate into different types of neural cells in the nervous system and play a vital role in nervous system development and neural damage repair^63^. NSC-derived extracellular vesicles (NSC-EVs) are rich in a number of specific miRNAs that participate in a variety of neurophysiological and pathological processes, including neurogenesis, neural regeneration, neuroinflammation, neuroprotection, and blood‒brain barrier maintenance^64^. NSC-EV-based therapy has been used mainly for the treatment of neural injury and degenerative diseases, including stroke, spinal cord injury, Alzheimer’s disease, and Parkinson’s disease. The specific therapeutic mechanism may involve regulating the activation and apoptosis of various types of cells in the tissue microenvironment, including endogenous NSCs, neurons, astrocytes, oligodendrocytes, microglia, and vascular endothelial cells^64,65^. Because NSC-EVs are derived from neural cells, they may provide therapeutic advantages over MSC-EVs in the treatment of neurological diseases, although there is no direct evidence that NSC-EVs are more effective than MSC-EVs obtained from other tissues. In terms of clinical applications, human NSCs may be limited. Human NSCs are currently derived mainly from aborted fetal neural tissue or differentiated induced pluripotent stem cells, both of which present great challenges in industrial production.

##### Endothelial progenitor cell-derived EVs

Endothelial progenitor cells (EPCs) are a type of stem cell that mainly originates from the bone marrow. EPCs migrate from the bone marrow through circulating blood to a site of injury caused by trauma, ischemia, or hypoxia, where they participate in tissue repair and regeneration^66,67^. EPC-derived extracellular vesicles (EPC-EVs) inherit the biological functions of EPCs, including inhibiting inflammation, promoting cell proliferation and angiogenesis, and inhibiting apoptosis. EPC-EVs have been used in animal models as therapeutic drugs for ischemic stroke, myocardial infarction, diabetes, and acute kidney injury, and as drug delivery tools for neural and bone repair^68^. EPC-EVs may provide advantages over MSC-EVs for promoting angiogenesis because EPC-EVs express certain EPC-specific markers (e.g., CD31, CD34, and VEGFR2) and are rich in angiogenesis-related mRNAs and miRNAs found in EPCs^69^. However, clinical translation of EPC-EVs may be limited because of their source and production capacity. Human EPCs are mainly isolated and cultured directly from human peripheral blood and umbilical blood, and large-scale culture technology for EPC-EVs needs to be developed to support their clinical use.

##### Immune cell-derived EVs

Immune cells are diverse and have many functions. EVs derived from immune cells in vivo are generally both immune-activating and immune-inhibiting, and their specific functions depend on the type of cells from which they originate and the physiological state of the target cells. For example, EVs can induce the immune suppression of dendritic cells (DCs) to inhibit apoptosis and induce the immune activation of DCs against viruses^70^. On the basis of their biological role in vivo, numerous studies have used various types of immune cells derived from EVs for tumor therapy, including DCs, macrophages, neutrophils, mast cells, B cells, T cells, and natural killer cells^71,72^. Immune cell-derived EVs can act on the same type of cell from which they are derived and on other types of immune cells and tumor cells, which can activate an antitumor immune response and inhibit tumor growth^72,73^. In general, immune cell-derived EVs promote tumor antigen presentation by antigen-presenting cells—such as macrophages, DCs, and B cells—enhance the response of helper T cells, and promote the killing activity of T cells and NK cells against tumor cells^73^. Immune cell-derived EVs have potential for use in tumor therapy, but there are many limiting factors in their clinical translation. For example, immune cells are obtained primarily from healthy donors. Thus, only small quantities of cells can be collected, and subsequent large-scale culture is difficult. In addition, the surfaces of immune cell-derived EVs are rich in a variety of immune function-related molecules; thus, special attention should be given to these EVs to ensure immune safety in clinical applications.

#### Milk-derived EVs

Milk-derived EVs (MI-EVs) are the most widely studied and clinically applicable EVs among all body fluid sources. According to currently published studies, the sources of MI-EVs are raw milk or dairy products from various mammals, including humans, cattle, pigs, sheep, and horses, among which bovine milk accounts for the vast majority^74^. MI-EVs are secreted by various types of cells in the mammary gland, including adipocytes, epithelial cells, stem cells, and immune cells. Compared with EVs derived from a single type of cell cultured in vitro, MI-EVs may be more abundant in proteins, nucleic acids, and bioactive molecules^75,76^. Therefore, MI-EVs have been widely studied recently as potential therapeutic products for disease treatment^74^.

EVs derived from milk have antitumor, anti-inflammatory, antioxidative, antifibrotic, and tissue regenerative effects^74,77^. EVs from milk have been shown to decrease tumor cell proliferation and increase responsiveness to chemotherapy drugs^78^. The anti-inflammatory and antioxidant effects of EVs derived from milk are reflected in their protective effects on intestinal inflammatory diseases, such as necrotizing enterocolitis and inflammatory bowel disease. By inhibiting the proliferation of hepatic stellate cells and alleviating cardiac fibrosis by promoting angiogenesis, milk-derived EVs inhibited fibrosis during recovery from liver injury^79,80^. In skin, hair, and bone injury-related diseases, EVs promote mainly tissue regeneration and repair by increasing target cell proliferation^74^. Engineered MI-EVs have been studied for the treatment of a variety of cancers, including lung, intestinal, breast, oral, and ovarian cancers^76^.

Compared with EVs derived from mammalian cell cultures, MI-EVs have several unique advantages but also application limitations^74,77^. The source of MI-EVs is highly abundant, and large-scale cell culture is not needed. MI-EVs originate from a variety of cell types and are rich in bioactive proteins and nucleic acids. Furthermore, MI-EVs have high stability and can withstand proteases at low pH values and high temperatures, indicating that MI-EVs can be delivered as oral drugs. However, the clinical translation of MI-EV applications is limited by the need for large-scale processes to collect EVs from animal milk, and because the use of animal components must be strictly controlled during drug production. Furthermore, it is difficult to realize genetic engineering on the basis of the endogenous loading of animal milk EVs (see the Engineering EVs section below).

#### Plant-derived EVs

Plant-derived EVs (P-EVs) are generally extracted from the tissue lysates of edible plants, such as fruits and vegetables^81,82^. P-EVs function in both physiological and pathological processes in plants, such as promoting cell proliferation and differentiation, remodeling the cell wall, and resisting pathogens^83,84^. P-EVs contain bioactive lipids, proteins, nucleic acids, and other biological macromolecules and a variety of natural phytochemicals with pharmacological activities, such as carotenoids and curcumin^85,86^. Owing to their natural biological properties, P-EVs have been widely used in pharmaceutical research.

As bioactive drugs, P-EVs have therapeutic effects on various human diseases. Preclinical studies have shown that P-EVs have antitumor, anti-inflammatory, antioxidant, and tissue regeneration activities^81–83^. P-EVs were shown to directly inhibit tumor cell cycle progression and tumor cell proliferation and promote tumor cell apoptosis; this was accomplished by inducing both reactive oxygen species production and proinflammatory factor release and by activating the apoptosis-inducing ligand pathway. P-EVs can also be combined with chemotherapy drugs to reduce tumor cell drug resistance^81^. Owing to their anti-inflammatory, antioxidant, and tissue repair-promoting effects, P-EVs have shown good therapeutic effects on liver injury, skin injury, gastrointestinal inflammation, and other diseases^82,83^. P-EVs have also been used as drug delivery vehicles for disease treatment^86^. For example, cabbage-derived EVs loaded with miR-184 and doxorubicin were used for tumor treatment^87^, ginger-derived EVs loaded with gingerol were used for treating ulcerative colitis^88^, and orange-derived EVs were used in SARS-CoV-2 mRNA vaccine research^89^.

Compared with EVs derived from cultured mammalian cells, P-EVs have both advantages and disadvantages. Their advantages include a more abundant source, no need for upstream cell production, a less extensive upstream production process, lower cost, no cytotoxicity, and lower immunogenicity. P-EVs are rich in a variety of natural plant metabolites and can be more effectively engineered to maintain the stability of external molecules, with more efficient release in vivo and better drug bioavailability. However, P-EVs have disadvantages, including the following: the target specificity of tissue cells in vivo must be verified; the downstream isolation process is complicated and costly; and it is difficult to realize genetic engineering on the basis of endogenous loading, similar to that of animal milk EVs. In general, although the current research on P-EVs is far less advanced than that on mammalian EVs, P-EVs are receiving increasing amounts of attention.

### Engineered EVs

Although natural EVs have potential as new drugs, they also have limitations, such as poor loading of specific molecules and insufficient targeting. Engineered EVs refer to EVs obtained by engineering modifications to source cells or natural EVs. Engineered EVs are designed to increase the ability of natural EVs to load the desired therapeutic drugs and improve their targeting properties through genetic, physical, or chemical means. Engineered EVs act directly as therapeutic drugs or as drug delivery vehicles. Some excellent reviews have introduced the latest progress in engineering EV drugs^90,91^. We briefly summarize the research and development of engineered EVs, which have focused mainly on loaded cargo and loading strategies (Table 3).

**Table 3:** Summary of engineered EVs: Load contents and loading methods

Engineered EV contents				
Category	Contents	Loading method	Loading purpose	Therapeutic effects
Disease-targeting contents	- Small-molecule drugs (e.g., curcumin, paclitaxel, doxorubicin) - Nucleic acids (siRNA, miRNA, mRNA) - Proteins (e.g., CD24)	-Exogenous loading -Endogenous loading	Therapeutic cargo loading	- Enhanced therapeutic effects (EVs as a drug delivery system) - Enhanced therapeutic efficiency (EVs as a natural drug)
Tissue and cell-targeting contents	- Proteins (e.g., peptides, antibodies) - Chemicals (e.g., azide and alkyl groups) - Biomaterials (e.g., Polyethylene glycol, hydrogels)	-Exogenous loading -Endogenous loading -Material-based loading	Tissue and cell targeting, controlled release	- Improved tissue and cell specificity - Enhanced tissue retention - Improved therapeutic efficiency

Engineered EV loading methods				
Category	Process	Methods	Contents	Advantages
Exogenous loading	Loaded directly on EVs, loading after EVs are purified from cells (postloading)	- Electroporation - Incubation - Sonication - Freeze–thaw - Extrusion - Transfection	- Small-molecule drugs - Small nucleic acids (siRNA, miRNA) - Chemicals - Biomaterials	- High loading efficiency - Fast process - Flexible drug combinations
Endogenous loading	Loaded directly on cells, loading before EVs are purified from cells (preloading)	- Transient expression - Incubation - Transfection - Epigenetic modifications - Long-term expression - Virus transfection - Genome editing	- Proteins (peptides, antibodies, enzymes) - Small and large nucleic acids (miRNA, mRNA, lncRNA)	- Genetic precision - Stable expression - Large-scale production
Material-based EV engineering	Loaded directly on EVs (postloading)	- Surface-based modifications - Chemical - Electrostatic - Magnetic - Scaffold-based modifications - Hydrogel - Microsphere - Microneedle	- Chemicals - Biomaterials	- Prolonged circulation - Enhanced tissue targeting - Sustained release - Local accumulation - Improved stability

*EVs* extracellular vesicles

#### Engineered EV loads

Natural EVs can be engineered to achieve or enhance their efficacy in treating specific diseases. The engineering of EVs depends primarily on the type of load they carry. On the basis of their biological characteristics, engineered EVs can be loaded with components such as small-molecule drugs, proteins, nucleic acids, and lipids.

##### EVs can be loaded with specific disease-targeting drugs

EV-loaded small-molecule drugs are primarily used in tumor therapy. Studies have shown that small-molecule drugs, such as curcumin, paclitaxel, and doxorubicin, have stronger inhibitory effects on tumors when encapsulated in EVs than free drugs do^92,93^. Many studies have focused on EV-loaded nucleic acid molecules. RNA-based gene therapy has been widely used in infectious, immune, genetic, and inflammation-related diseases and cancer^94^. The RNA molecules loaded in EVs can be divided into two categories^95^. The first comprises small RNA molecules that regulate gene expression, such as small interfering RNAs (siRNAs), antisense oligonucleotides, and oligonucleotides that can recruit endogenous RNA-editing enzymes^96^. These miRNAs are relatively simple to load into EVs and can be synthesized with chemical modifications at specific residues to alter their stability, pharmacokinetics, and potential to elicit an immune system response. The second category consists of therapeutic mRNAs with a relatively large molecular weight that can be translated into proteins with therapeutic activity or into antigens that induce an immune response that fights or prevents disease (e.g., mRNA vaccines)^97^. Compared with liposome-based nanoparticles, engineered EVs can deliver mRNAs to specific sites, avoid recognition and early degradation by the immune system, overcome biological barriers (e.g., the blood–brain barrier), and control drug release under specific external stimuli^98,99^. Proteins can also be loaded into EVs^100^. Representative results were obtained from a series of studies on CD24, which binds to damage-associated molecular patterns to inhibit inflammatory responses mediated by NF-κB pathway activation^101,102^. EVs loaded with CD24 have shown safety and efficacy in mouse models of various lung diseases, including sepsis, allergic asthma, chronic obstructive pulmonary disease, and pulmonary fibrosis, and a series of clinical studies have confirmed their safety and efficacy in the treatment of COVID-19-associated acute respiratory distress syndrome (ARDS)^102,103^.

##### Natural EVs can be modified to enhance tissue and cell targeting and loaded with specific drugs to enhance their therapeutic effect

Natural EVs are rich in various bioactive molecules that can regulate cell behaviors after being absorbed by tissues and cells. Although they have potential for treating various diseases, their ability to target the body is insufficient; therefore, their therapeutic effect is limited. Protein and polypeptide modifications of natural EVs can greatly improve their targeting and therapeutic abilities^104^. EVs with enhanced function and targeting ability can be engineered by fusing peptides with targeting functions to EV membrane surface proteins, as well as by loading EVs with drug molecules specific to a disease^105^. Engineered EVs have been used in preclinical studies to treat various diseases. For example, loading cysteine–arginine–glutamate–lysine–alanine (CREKA) peptides onto the surface of EVs produced engineered EVs that targeted the fibrin-fibronectin complex via CREKA peptides, which increased retention of the EVs in rat femur defect sites and enhanced bone repair in rat femur defect models^106^. The highly expressed interleukin-3 (IL3) receptors in chronic myeloid leukemia (CML) cells can serve as drug targets. IL3 and the EV scaffold protein LAMP2B were fused to construct engineered LAMP2B-IL3 EVs, and siRNAs targeting CML cell oncogenes and the chemotherapeutic imatinib were loaded simultaneously to generate engineered EVs that targeted CML cells, inhibited cancer cell growth and reduced tumor size^107^. Rabies virus glycoprotein (RVG) peptides bind selectively to acetylcholine receptors on neural cells. RVG peptides and the EV scaffold protein LAMP2B have been fused to construct engineered LAMP2B-RVG EVs, and along with miRNA-124, which exerts a neuroprotective effect, they have been loaded into EVs. The engineered EVs penetrated the blood–brain barrier and ischemic area of the cortex and promoted neurogenesis^108^.

#### Engineering EV loading methods

Drugs can be exogenously or endogenously loaded into EVs^109^. Exogenous drug loading is also referred to as postloading, in which EVs from cell culture or other biological fluids are isolated and purified and then functional molecules are loaded onto the surface or inside the EVs by physical or chemical means. Endogenous drug loading is also referred to as preloading, in which donor cells are bioengineered to produce EVs containing specific drugs.

##### Exogenous drug loading of EVs

Exogenous drug loading of EVs involves the direct introduction of drugs into the EVs^110^. Common methods include the following: (i) electroporation, which forms temporary pores in the EV membrane via high-voltage pulses to allow drug penetration and is suitable for loading small molecules and RNA, but high-voltage pulses may cause siRNA precipitation and EV aggregation; (ii) incubation, in which EVs are mixed with a drug under specific conditions so that the drug enters the EV driven by a concentration gradient; (iii) ultrasound, in which drugs enter through the action of sound waves; this is suitable for loading small RNA molecules and does not cause RNA aggregation or degradation; (iv) freeze–thaw cycling, which disrupts EV membranes by repeated freezing and thawing, allowing drugs to enter the vesicle but may destroy membrane structures; (v) extrusion, which uses special equipment to pass EVs through narrow channels to increase membrane permeability and allow drugs to enter; and vi) chemical transfection, which is often used to load nucleic acids through chemical or electrical means. The best method for exogenous drug loading depends on the EV source, cargo, and intended application, and a better presentation of the detailed introduction of these methods can be found in some recent reviews^90,91^.

Compared with endogenous loading, exogenous loading has advantages and disadvantages^111^. The main advantage of exogenous loading is the significantly greater drug loading efficiency than that of endogenous loading. The disadvantages of exogenous loading chiefly include changes in or even destruction of the physical and chemical factors of EVs, the loss of EVs caused by repurification after drug treatment, and reduced EV drug purity caused by the presence of exogenous substances. More importantly, the precise modifications of EVs that can be attained through genetic engineering are not possible through exogenous loading.

##### Endogenous drug loading of EVs

Endogenous drug loading of EVs is based mainly on genetic engineering technology, in which functional nucleic acids and protein molecules are expressed in source cells, where they are loaded into the EVs produced by the cells. The EVs that are harvested from the cell culture supernatant are therefore already loaded with the expressed molecule. Genes can be introduced into recipient cells via either nonviral transient transfection or virus-based long-term transduction. Nonviral transfection typically results in high transient expression, but the cells cannot be subcultured to obtain drug-loaded EVs continuously, whereas viral transfection results in low transient expression, but the cells can continuously produce drug-loaded EVs. The two methods have their own advantages and disadvantages and therefore have specific application scenarios.

Exogenously loaded EVs have greater drug loading efficiency than endogenously loaded EVs do, but the latter can be engineered with many more detailed and precise modifications. Some studies have made full use of the advantages of genetic engineering to produce engineered EVs with high drug loading efficiencies that act as targeted, multifunctional drugs. In what follows, we provide a summary of several major genetic engineering techniques used for EVs.

*A. Fusion expression of specific proteins and EV component proteins to promote protein loading*

To address the low loading efficiency of the desired protein, a fusion protein containing the protein of interest and an EV component protein or scaffold protein can be constructed, in which the fusion protein enters the EV under the guidance of the component protein to improve the drug loading efficiency and EV targeting. In addition to the membrane proteins enriched in EVs that serve as markers (e.g., LAMP2B and CD63), other EV scaffold proteins can also be used in fusion proteins, such as PTGFRN and BASP1, which have been newly identified via proteomic analyses^112^. Studies have shown that specific proteins, such as cytokines, antibody fragments, RNA-binding proteins, vaccine antigens, and Cas9 proteins, can be efficiently loaded onto the surface of EVs by fusing them with PTGFRN or moving them into the lumen of EVs through fusion with BASP1^112–115^.

*B. Expression of guide peptides promotes tissue and cell targeting of EVs*

Once the guide peptide fused with the EV surface protein is expressed, the guide peptide can deliver the EVs to specific tissues and cells in vivo^105^. LAMP2B is a classic guide peptide example, with LAMP 2B-IL3EV targeting CML cells^107^, LAMP2B-RVGEV targeting nerve cells^108^, LAMP2B-iRGDEV targeting breast cancer cells^116^, LAMP2B-tLyP-1EV targeting non-small cell lung cancer cells^117^, and LAMP2B-CAPEV targeting chondrocytes^118^. These genetically modified EVs combined with specific drug molecules have shown strong therapeutic effects in animal models of disease.

*C. Selective loading of functional miRNAs into EVs via EXO motifs*

EVs from different cell sources contain different endogenous miRNAs. The miRNAs present in natural EVs are actively encapsulated, and their biological processes are finely regulated. Previous studies have suggested that miRNAs are localized to EVs in different cell types through cellular localization motifs (CELL motifs) and extracellular vesicle localization motifs (EXO motifs)^119^. Recent studies have demonstrated that cell type-specific EXO and CELL motifs, along with the RNA-binding proteins Alyref and Fus, are involved in the selective loading of miRNAs^120^. These motifs act as sorting codes for miRNAs, and parsing these codes is key to achieving fine-tuned regulation of EV-derived miRNAs. For example, in hepatocytes, a common miRNA sorting sequence (GGCU, an EXO motif) abundant in EV-miRNAs binds to the RNA-binding protein SYNCRIP and controls the sorting of these miRNAs into EVs. Embedding EXO motifs in miRNAs enhances the loading of these miRNAs into hepatocyte-derived EVs. The understanding of this finely regulated loading mode is still in its infancy, and additional research is crucial for the selective loading of miRNAs into EVs for future applications.

*D. Epigenetic approaches to EV modifications*

Emerging evidence has highlighted epigenetic modulation as a promising alternative to genetic engineering for tailoring EVs. By altering the epigenetics of donor cells (e.g., DNA methylation, histone modifications, and noncoding RNA regulation), EV cargo composition and functionality can be dynamically reprogrammed without the risk of genomic integration. For example, by enhancing HDAC inhibition, tumor-suppressing miRNAs become enriched in EVs, thereby increasing EV antitumor efficacy^121^. Epigenetic modifications can also be reversible and render stimulus-responsive control, providing spatiotemporal control over EV release under conditions such as hypoxia or drug treatment^122^. While epigenetic effects may be less persistent than genetic modifications are, epigenetic strategies can be a safer route for clinical translation by preventing permanent genomic alterations.

#### Material-based EV engineering

The distribution of drug-loaded EVs in vivo without any surface modifications is similar to that of natural EVs. However, engineering EVs with specific surface modifications (e.g., targeting peptides) can increase their ability to target specific tissues and cells. In addition to genetic engineering, a variety of materials have been developed on the basis of the physical and chemical characteristics of EVs, which promote EV targeting and enhance their therapeutic effects^123–125^. However, despite targeting, EVs may be rapidly cleared before they reach the target tissue due to the defense mechanisms of the immune system and filtration by the liver and kidney. Furthermore, the natural characteristics of EVs cause them to be rapidly absorbed in the body; therefore, it is difficult for EVs to accumulate and persist in local tissues^126,127^. To address these problems and strengthen the therapeutic effects of EVs, researchers have developed materials-based EV engineering technologies that enhance tissue and cell targeting, provide sustained and controlled EV release, and improve drug delivery efficiency.

##### EV surface-based material engineering

EV surface covalent chemical modifications: Transmembrane and extracellular proteins on the surface of EVs carry abundant carboxyl (–COOH), amino (–NH2), and sulfanilamide (–SH) groups. Thus, azide or alkyl groups can be added to the EV surface through condensation to create active chemical sites that can further couple various targeting structures via click chemistry in different buffers, including water, dimethyl sulfoxide, and alcohol^128,129^. This method is well suited for covalently bonding small molecules, macromolecules, and polymers to EV surfaces. Compared with traditional chemical reactions, click chemistry is more efficient and enables stronger control over binding sites, which helps to connect specific structures and therapeutic drugs to EVs and has been shown to enhance tissue and cell targeting for treating central nervous system injuries and acute liver failure^130–132^.

Noncovalent physical modifications of the EV surface: These methods use mainly electrostatic and hydrophobic interactions to load cargo onto the EV surface^128^. Electrostatic modifications use the principle of attraction between different charges. For example, the fusion of cationic lipids, such as their encapsulation by dextran macromolecules, positively charges the EV surface and increases the efficiency of EV intercellular uptake and intracellular release. Hydrophobic interactions can efficiently and spontaneously integrate lipophilic substances into the EV membrane. For example, cyclic RGD was linked to the terminal end of a conjugate of 1,2-dioleoylglycerol-3-phosphoethanolamine and polyethylene glycol, which, when incubated with EVs, was automatically inserted into EV membranes. These modified EVs exhibited significantly increased binding specificity to tumor cells^133^.

EV surface magnetic modifications: Tissue-targeted delivery can also be achieved by modifying EVs with magnetic nanoparticles. Studies have shown that EVs anchored with superparamagnetic iron oxide nanoparticles and tumor necrosis factor can both enable tumor targeting under the action of external magnetic fields and inhibit tumor growth^134^. Some researchers loaded magnetic nanoparticles onto the surface of EVs containing doxorubicin, guided the EVs to the tumor site via an external magnetic field, and then induced local hyperthermia with near-infrared radiation to trigger the EVs to release their drug cargo, providing a new approach for the precision treatment of tumors^135^.

##### Scaffold-based EV engineering

The incorporation of EVs into scaffold systems enables targeted and site-specific delivery, prolongs the EV half-life, and reduces degradation rates, thereby increasing the therapeutic persistence and bioactivity of the EVs. Studies have shown that scaffold-mediated EV delivery effectively promotes tissue remodeling, wound healing, bone regeneration, immune modulation, and angiogenesis; therefore, it is an increasingly popular focus of tissue engineering research^136^. There are several ways to use EVs in scaffold systems, as listed below.

Hydrogel-based engineering: Hydrogels are hydrophilic three-dimensional network gels that can absorb many times their weight in water without destroying their structure^137^. The advantages of using hydrogels include biocompatibility, degradability, and swelling. Hydrogels can be used as cell support materials in tissue repair and regeneration processes and as efficient carriers for drug delivery^138^. Studies have shown that hydrogel-EV biocomplexes can protect EVs from immune clearance in vivo and slow their release, increasing the tissue exposure time to the drug and improving the healing effect of EVs in various types of tissue injury^139,140^.

Nanoparticle- and microsphere-based engineering: Microsphere-based biomaterials have been used as injectable microscaffolds to deliver EVs^123^. A platform capable of efficiently delivering EVs with slow release kinetics into irregularly shaped defect bones was developed^141^. The platform was based on a simple adsorption technique in which polydopamine (PDA) was coated onto injectable porous polylactide-co-glycolide (PMS) microspheres to form PMS-PDA microspheres, and EVs were effectively adsorbed onto the microspheres. The PMS-PDA microspheres maintained high activity and induced vascularized bone regeneration in rat skull defects^141^.

Microneedle-assisted delivery: Microneedle delivery is an advanced drug delivery technology that uses microneedle patches as carriers to efficiently deliver EVs to target sites^142^. Compared with traditional injection systems, microneedle patch delivery is painless and allows efficient local delivery of EVs to target sites to achieve the desired therapeutic effects^91^. Currently, microneedle-based EV delivery systems have been developed for hair regeneration, skin wound healing, spine cord injury, and plasmacytoma^91,143,144^. Microneedles also serve as a long-term storage method for EVs. In one study, EVs in hyaluronic acid microneedles were stored at 4°C for 6 months^145^.

### Overview of basic and clinical studies on EV drugs

**Fig. 2:**
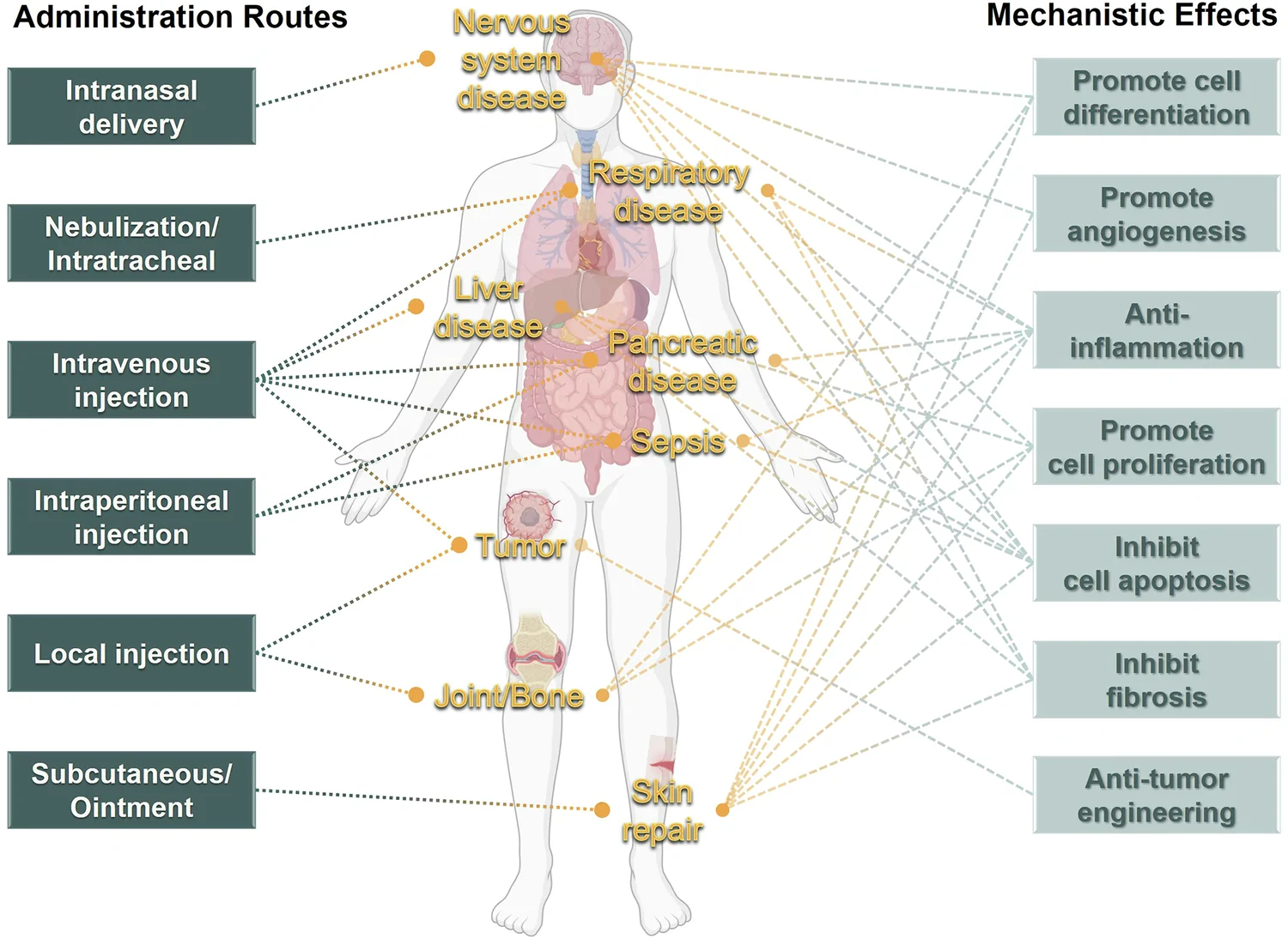
Therapeutic applications of extracellular vesicles from various systems in treating diseases. The figure summarizes the diverse clinical applications of extracellular vesicle-based therapies, organized by administration routes, target diseases, and mechanistic effects. The dashed lines indicate the administration routes and the mechanistic effects of the target disease. The representative schematics in this figure were created with BioRender.com

Compared with traditional drugs, EV drugs have multiple advantages^146,147^. EVs are multifunctional, as they can be designed to carry a number of different therapeutic molecules to enhance their therapeutic effects and increase the overall efficacy of treatment. EVs can overcome the limitations of traditional delivery systems by crossing biological barriers, such as the blood–brain barrier, to reach lesions that can be difficult to access via traditional drugs. EVs exhibit enhanced targeting through specific surface molecules on target cells to accurately deliver therapeutic molecules to target tissue, which helps to concentrate the therapeutic effect at the target site while reducing off-target effects on normal tissues. EV drugs also have advantages over cell therapy products^148^. EVs cannot replicate themselves, which eliminates the risk of abnormal cell differentiation, immune rejection, or tumor formation. In addition, EVs are relatively small, have a simple structure, and are therefore relatively simple to produce and store at a large scale, which is convenient for clinical application. As a result, EV drugs have been used for treating many diseases in basic research and at the clinical stage (Fig. 2). Basic and clinical studies of EV drugs used against major diseases are summarized below.

#### Respiratory diseases

Respiratory diseases kill millions of people worldwide every year. Despite advances in our understanding of the pathogenesis of respiratory diseases and advances in modern medicine and drug design, the main available treatments remain limited to treating symptoms or delaying disease progression. Most existing drugs primarily limit disease progression or prevent complications. Recent studies have shown that EV-based therapies, particularly MSC-derived EVs, are expected to provide novel strategies for treating respiratory diseases.

Bone marrow, adipose tissue, human menstrual blood, and umbilical cords are the primary sources of MSCs. The therapeutic effects of MSC-EVs on respiratory diseases include reducing inflammation, cell death, and oxidative stress and preventing epithelial‒mesenchymal transition^149^. MSC-EVs primarily affect cell behavior and disease progression by delivering regulatory miRNAs that target specific signaling pathways in target cells. MSC-EVs carry more than 40 different miRNA species, producing favorable therapeutic effects in animal models of different respiratory diseases, including acute lung injury (ALI), pulmonary ischemia/reperfusion (I/R) injury, idiopathic pulmonary fibrosis (IPF), radiation-induced lung injury, sepsis-induced acute lung injury, hyperoxia-induced lung injury (HILI), and other diseases, such as pulmonary fibrosis silicosis, asthma, and coronavirus disease (COVID-19). For example, MSC-derived EVs downregulated STAT3 via miR-125b-5p, which inhibited macrophage pyroptosis and alleviated sepsis-associated ALI^150^. EVs transfer miR-let-7 into MLE-12 cells to inhibit the expression of Sp3, weaken the recruitment of Sp3 to HDAC2, relieve the deacetylation restriction of HDAC2 to Nrf2, and enhance Nrf2 pathway activation, which reduces iron death signaling in the cells and delays the pathological process of oxidative damage and apoptosis of lung epithelial cells in an IPF model^151^. MSC-EVs have been shown to regulate classical signaling pathways, such as the PI3K/Akt, NF-κB, Nrf2, PTEN, Wnt, MAPK, Toll-like receptor, and AMPK pathways, through a variety of other miRNAs to reduce oxidative stress, regulate the immune response, reduce the expression of inflammatory cytokines, and promote tissue repair^152^.

#### Nervous system diseases

Traumatic and degenerative diseases of the nervous system, such as stroke, spinal cord injury, Alzheimer’s disease, and Parkinson’s disease, continue to pose great challenges to modern medicine. The main pathogenic mechanism of these diseases is the death of functional neurons induced by injury or toxic molecules accompanied by nervous system inflammation, which gradually leads to motor or cognitive dysfunction in patients. Currently, effective clinical treatments are very limited. An increasing number of studies have been conducted using EV drugs to treat nervous system diseases, and their effectiveness in neural protection and repair has been demonstrated^16,153^. The neural protection and repair effects of EV drugs are reflected at three levels: at the tissue level, EVs promote neural regeneration and reduce neuro-related muscle atrophy; at the cellular level, EVs promote neuronal survival and reduce inflammatory astrocyte and microglia activation; and at the molecular level, EVs reduce proinflammatory factors, increase neurotrophic factors, and reduce the deposition of toxic protein particles in neurons, such as amyloid protein.

Early studies on basic stroke have shown that platelet-derived EVs increase neural stem cell proliferation, neurogenesis, and angiogenesis in the ischemic brain in a dose-dependent manner and that monocyte-derived EVs induce neuroprotective effects^154,155^. More recent studies have used natural or engineered MSC-EVs to treat stroke. For example, natural EVs from bone marrow and adipose MSCs reduce inflammation and neuronal death and promote neural regeneration, angiogenesis, and synaptic remodeling, thus improving the neurological function of stroke model animals^156,157^. Engineered MSC-EVs overexpressing miRNAs, including miR-133b, miR-17-92, and miR-181b-5p, significantly enhanced brain plasticity after stroke and promoted neurological recovery^158,159^. RVG peptide-LAMP2B-miRNA-124-engineered MSC-EVs effectively delivered EVs to ischemic brain regions to reduce brain damage specifically by delivering miRNA-124 to cells to promote neural progenitor cell differentiation^108^.

Amyloid-β (Aβ) deposition is a major pathogenic factor of Alzheimer’s disease^160^. Natural EVs from neuronal cells accelerated the clearance of Aβ in an Alzheimer’s disease mouse model, which reduced synaptic neurotoxicity and inflammatory factor levels and improved the learning and memory ability of the mice^161,162^. Engineered neuronal cells overexpressing Fe65 and loaded with corynoxine-B (an autophagy inducer) targeted neurons expressing Aβ to induce autophagy, thus alleviating the pathological progression of Alzheimer’s disease^163^. Similarly, natural and engineered MSC-EVs reduce Aβ levels and proinflammatory factors and promote neurogenesis, which improves cognitive function in patients with Alzheimer’s disease^164–166^. Using a similar therapeutic mechanism, EVs are also being extensively studied for use in other neurological diseases, such as amyotrophic lateral sclerosis, Huntington’s disease, multiple sclerosis, and spinal cord injury^167^.

#### Severe acute inflammatory diseases

Severe acute inflammation-related diseases, such as acute liver failure (ALF), severe acute pancreatitis (SAP), and sepsis, are characterized by acute inflammation, cumulative multiorgan damage, and high mortality. Reducing inflammation and tissue damage and promoting tissue repair are fundamental to the successful treatment of these diseases. On the basis of their natural anti-inflammatory and tissue repair effects, a growing number of preclinical studies have explored the therapeutic potential of MSC-EVs in these diseases^168,169^.

MSC-EVs from various tissues have been shown to effectively treat ALF through the action of specific molecules. For example, adipose MSC-EVs act on liver cells through the long-chain noncoding RNA (lncRNA) H19, which reduces liver damage and inflammation levels and improves survival rates in ALF model rats^40^. Adipose MSC-EVs were also shown to inhibit TXNIP/NLRP3-mediated activation of inflammatory bodies through the action of miR-17 on inflammatory macrophages, which alleviated the symptoms of ALF model mice^170^. Both bone marrow and umbilical cord MSC-EVs protect ALF hepatocytes by exerting antiapoptotic effects^171,172^. Engineered placental MSC-EVs that were designed to specifically target the liver exhibited high efficacy in the ALF model^132^.

Previous studies have shown that treatment with MSC-EVs from SAP patients protects pancreatic acinar cells through metabolites. For example, hypoxia-induced umbilical cord MSC-EVs delivered functionally active mitochondria, and TNF-α-induced umbilical cord MSC-EVs delivered functional metabolites, such as dihydroxyphenyl glycol. These active metabolites inhibited pancreatic acinar cell damage and reduced tissue inflammation, thereby alleviating SAP progression^173,174^. MSC-EVs also reduce myocardial injury induced by SAP through different mechanisms: bone marrow MSC-EVs downregulate the HMGB1/TLR4/Akt signaling axis, and human-induced pluripotent stem cell-derived MSC-EVs activate the Akt/Nrf2/HO-1 signaling axis in cardiomyocytes^175,176^.

Several preclinical studies have demonstrated the efficacy of MSC-EVs derived from different tissues for treating sepsis, including fat, bone marrow, and umbilical cord placenta^177^. The efficacy of MSC-EVs in treating sepsis is reflected by three metrics: improved survival rates; reduced tissue damage, such as in the lungs, liver, kidneys, and heart; and decreased levels of proinflammatory factors, such as TNF-α, IL-1β, and IL-6^177^. On the basis of extensive knowledge of the function of MSC-EVs, studies have also focused on exploring their efficacy for treating sepsis without elaborating on the specific molecular mechanisms involved. Few studies have evaluated miRNAs, such as miR-223 and miR-146, for use in MSC-EV-mediated immunosuppression in sepsis^178^.

#### Tumors

EVs play dual therapeutic roles in tumors. As intrinsic therapeutic agents with natural tumor-targeting ability, immune cell-derived EVs—such as those from DCs, macrophages, NK cells, and T/B cells—have been widely explored for tumor therapy, as they can target both their parent and other immune/tumor cells to trigger antitumor responses and suppress tumor growth^71–73,179^. As drug carriers with engineered tumor-targeting capabilities, EVs can deliver various antitumor drugs, including siRNAs, miRNAs, the CRISPR-Cas9 system, chemotherapy drugs, precursor drugs, and monoclonal antibodies, either alone or in combination, to tumor cells to directly induce apoptosis or inhibit proliferation^180,181^.

In basic studies, EVs encapsulating small-molecule drugs showed stronger tumor inhibitory effects than did the corresponding free drugs, as previously described^92,93^. Moreover, engineered EVs with membrane surface modifications can induce immune cells to target tumor cells in vivo for cell killing^182^. Antitumor drug delivery via engineered EVs improves drug targeting, reduces side effects, and enables the delivery of drug combinations, thus improving therapeutic efficacy^183^. LAMP2B-IL3-engineered EVs were simultaneously loaded with siRNA against oncogenes and the chemotherapeutic agent imatinib, thus targeting CML cells through the IL3 receptor and inhibiting their proliferation^107^. Engineered EVs loaded with CRISPR-Cas9 complexes achieved precise gene editing specific to liver cancer cells and effectively inhibited tumor growth^184^. EVs, such as engineered EVs that effectively deliver PD-L1-blocking antibodies to liver cancer cells, which significantly enhance the recognition and killing of liver cancer cells by T cells, thus improving tumor immunogenicity, have also been used in tumor immunotherapy^181,185^.

#### Progress in clinical trials of EV drugs

**Fig. 3:**
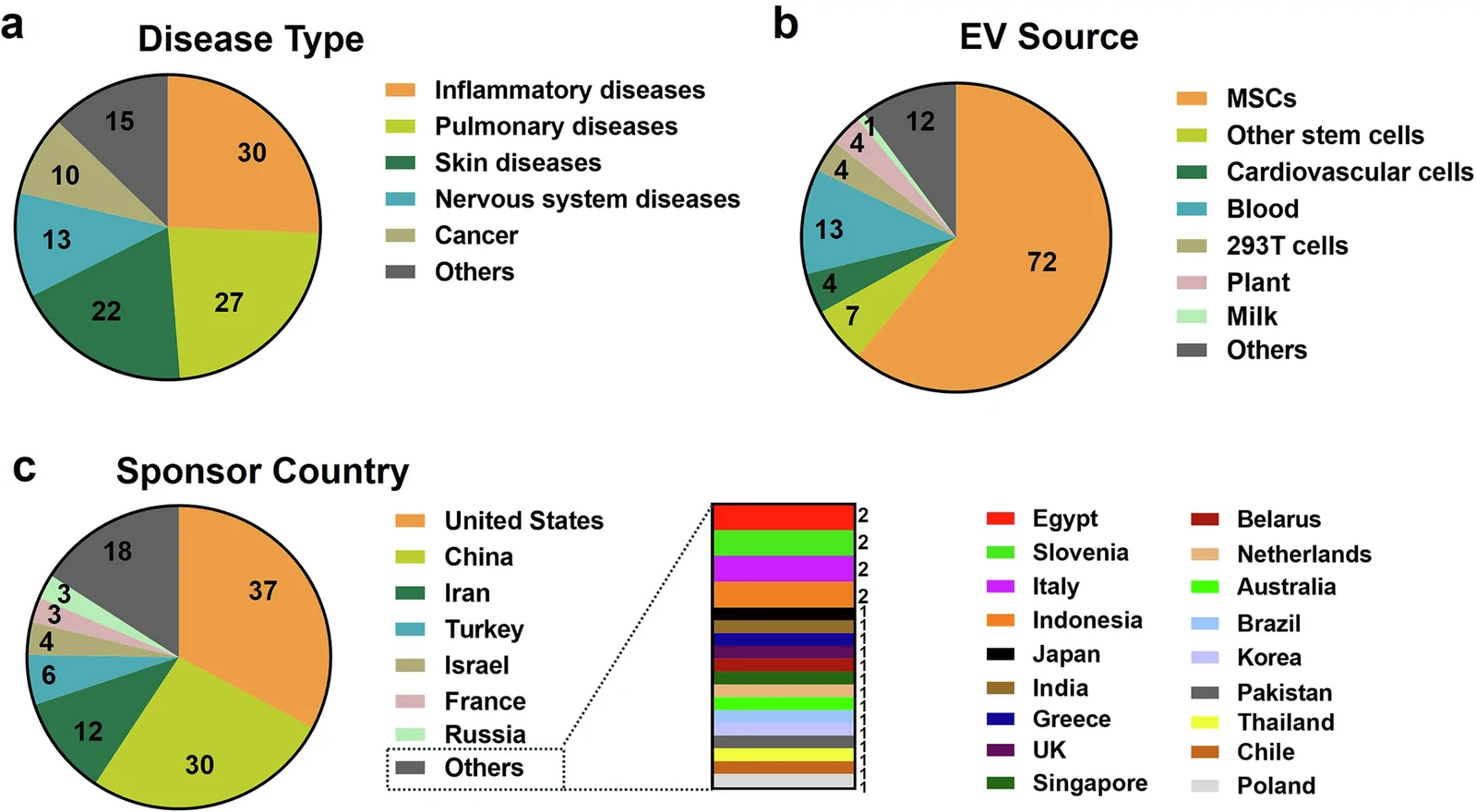
Statistical analysis of the disease type, extracellular vesicle (EV) source, and sponsor country of the 117 interventional studies. **a** The disease type was categorized into inflammatory (e.g., osteoarthritis, Crohn’s disease, ulcerative colitis, inflammatory bowel disease), pulmonary (e.g., COVID-19, acute respiratory distress syndrome, bronchopulmonary dysplasia), skin (e.g., burns, wounds, androgenetic alopecia, fistula perianal), nervous system (e.g., ischemic stroke, Alzheimer disease), cancer (e.g., lung cancer, colon cancer, lymphoma), and other diseases (e.g., premature ovarian failure, myocardial infarction). **b** The EV sources used in the studies were categorized. Other stem cells represent stem cells that are not mesenchymal stem cells, such as induced pluripotent stem cells and limbal stem cells. The blood source included blood cells and plasma. Others are sources that are not included in the categorized sources. **c** The sponsor countries of the studies were analyzed. The values represent the number of studies and are shown in Arabic numerals

Despite these challenges, advances in research and technology are pushing EV therapy to become an important part of modern medicine. EV drugs are still in clinical trials but have already shown great potential for treating diseases. As of January 2025, 292 EV-related clinical trials (“extracellular vesicle” or “exosome” as keywords for searching) were registered on Clinical Trials.gov, including 122 observational studies and 170 interventional studies. One hundred seventeen interventional studies were designed to assess the therapeutic effects of EV drugs across diverse disease areas, including inflammatory, pulmonary, skin, and nervous system diseases and cancers (Fig. 3A). The analysis indicated that while various sources are being used for EV drugs, MSCs are the most widely used ones (72/117, 61%) (Fig. 3B). The sponsor country analysis reflects concentrated research efforts in the U.S. and China, with increasing global participation (Fig. 3C). Most of these studies are in Phase I or II (85 studies, 73%) and use natural EVs (110 studies, 94%); only 7 studies (6%) use engineered EVs. We list promising EV-based therapy-related interventional studies (Table 4) and discuss several representative trials below.

**Table 4:** Promising extracellular vesicle-based therapy-related interventional studies

Candidate	Condition	Approval	Source	Engineered	Phase	Sponsor	NCT No.
ExoFlo(DB-2Q)	ARDS	U.S. FDA	bmMSCs	-	Phase 3	Direct Biologics, LLC	05354141
ExoFlo(DB-3Q)	Crohn’s disease	U.S. FDA	bmMSCs	-	Phase 1 Phase 2	Direct Biologics, LLC	05836883
ExoFlo(DB-3Q)	Crohn’s disease	U.S. FDA	bmMSCs	-	Phase 1	Direct Biologics, LLC	05130983
DB-3Y	Refractory ulcerative colitis	U.S. FDA	bmMSCs	-	Phase 1	Direct Biologics, LLC	05176366
AGLE-102	Dystrophic epidermolysis bullosa	U.S. FDA	bmMSCs	COL7 COL7A1	Phase 1 Phase 2	Aegle Therapeutics	04173650
AGLE-102	Burn wounds	U.S. FDA	bmMSCs	COL7 COL7A1	Phase 1	Aegle Therapeutics	05078385
BRE-AD01(BxC-I17e)	Atopic dermatitis	U.S. FDA	Stem cells	-	Phase 1	Brexogen Inc.	06055361
ILB-202	Inflammation	HREC (Australia)	-	superrepressor IκBα	Phase 1	ILIAS Biologics	05843799
Zofin	COVID-19	U.S. FDA	Human amniotic fluid	-	Phase 1 Phase 2	ZEO ScientifiX, Inc.	05228899
Ardoxso	COVID-19 and ARDS (coronavirus pneumonia)	U.S. FDA	MSCs	-	Phase 1 Phase 2	AVEM HealthCare	04798716
PEP	Diabetic foot ulcers	U.S. FDA	Platelets	-	Phase 2	Rion Inc.	06319287
PEP	Knee osteoarthritis	U.S. FDA	Platelets	-	Phase 1	Rion Inc.	06463132
EXOB-001	Bronchopulmonary dysplasia	U.S. FDA EMA(Europe)	ucMSCs	-	Phase 1 Phase 2	EXO Biologics S.A.	06279741
SNE-101	Stroke	MFDS (Korea)	ucMSCs	-	Phase 1b	S&E bio	NA
AB126	Acute ischemic stroke	U.S. FDA	Neural cells	-	Phase 1b/2a	Aruna Bio	NA
XoGloPro	COVID-19	U.S. FDA	Placenta MSCs	-	Phase 1a	Kimera Labs	NA

*ARDS* acute respiratory distress syndrome, *U.S. FDA* United States Food and Drug Administration, *HREC* Human research ethics committee, *EMA* europeanm medicines agency, *MFDS* ministry of food and drug safety*, bmMSCs Bone marrow mesenchymal stem cells, MSCs mesenchymal stem cells, ucMSCs Umbilical cord mesenchymal stem cells*

The clinical use of MSC-EVs in respiratory disease was initially promoted by their positive effects in patients infected with SARS-CoV-2 (the causative virus of COVID-19). Lung tissue damage caused by COVID-19 may progress to ARDS and lead to respiratory failure, which is the primary cause of death in critically ill patients. In May 2022, a study from Ruijin Hospital with collaborating groups determined that the use of adipose MSC-EVs for the treatment of severe COVID-19 disease via aerosol inhalation was acceptably safe (NCT04276987)^186^. In June of the same year, Nanjing Medical University reported that aerosolized EV drugs derived from umbilical cord mesenchymal stem cells (MSCs) demonstrated both safety and efficacy in treating mild COVID-19 pneumonia (ChiCTR2000030261). The study revealed no allergic reactions, along with accelerated absorption of pulmonary lesions and shortened hospital stays^187^. In December 2023, Direct Biologics announced favorable safety data and significant efficacy results from a phase 2 clinical trial (NCT04493242) of ExoFlo™ in hospitalized adult COVID-19 patients with moderate-to-severe ARDS. The data revealed a 30.8% absolute risk reduction (61.6% relative risk reduction) in 60-day mortality for the 15 mL ExoFlo cohort compared with the placebo cohort. Notably, the 18–65-year subgroup had a 41.9% mortality reduction^188^. In addition, Israeli researchers and collaborating teams have demonstrated the safety and efficacy of CD24-loaded EVs in >180 patients with COVID-19-associated ARDS in phase 1b/2a, phase 2b, and compassionate use (NCT04747574, NCT04902183, and NCT05947747)^103,189,190^. At present, more than 30 clinical trials of MSC-EVs for the treatment of COVID-19 infection, associated pneumonia (ARDS), and pulmonary fibrosis have been registered at the National Institutes of Health (NIH) (clinicaltrials.gov).

Many basic studies have promoted the clinical translation of EV drugs for treating nervous system diseases. In 2022, a team from Iran reported the first clinical safety study of EVs in patients with stroke. The results showed that EVs derived from allogeneic placental tissue did not cause serious adverse events within 3 months after intraparenchymal injection into patients with stroke^191^. On this basis, the team initiated a clinical trial to evaluate the safety and efficacy of intravenous injection of allogeneic bone marrow MSC-EVs engineered to deliver miR-124 for the treatment of acute ischemic stroke (NCT03384433). Researchers from the Shanghai Jiao Tong University School of Medicine in China initiated a clinical trial evaluating the clinical safety and efficacy of allogeneic adipose MSC-EVs for the treatment of mild to moderate Alzheimer’s disease (NCT04388982). The preliminary results demonstrated the safety of intranasal EV administration and suggested effective doses for clinical application, with further research ongoing^192^. Excitingly, in January 2024, the US Food and Drug Administration (FDA) approved Aruna Bio’s investigational new drug application for the drug candidate AB126, unmodified neural cell EVs. AB126 has been shown to cross the blood‒brain barrier and exert anti-inflammatory and neural protective effects; therefore, it has the potential to treat a range of neurodegenerative diseases. In May 2025, S&E bio received approval from Korea’s Ministry of Food and Drug Safety (MFDS) to initiate a phase 1b clinical trial of SNE-101, an EV drug from umbilical cord-derived mesenchymal stem cells, for its investigational stroke therapy.

Although MSC-EVs currently represent the majority of clinical EV research due to their inherent immunomodulatory and tissue-repair capabilities, existing COVID-19 therapies continue to face challenges with adverse effects, and neurodegenerative disorders still lack disease-modifying treatments. EV-based therapeutics could provide a paradigm-shifting solution, contingent upon additional validation. However, despite promising results, critical safety concerns persist—particularly regarding long-term risks such as miRNA-driven off-target activity and unintended organ biodistribution—necessitating further comprehensive study. Additionally, clinical studies of EVs for tumor therapy are currently being conducted^193^. These include EVs loaded with siRNAs that inhibit KRASG12D for the treatment of pancreatic cancer (NCT03608631), EVs loaded with tumor antigens for the treatment of non-small cell lung cancer (NCT01159288), and EVs loaded with STING agonists (CDK-002, exoSTING) in patients with advanced/metastatic, recurrent, injectable solid tumors (NCT04592484). As technology continues to advance and our understanding of EV biology deepens, EVs are emerging as promising therapeutic agents with expanding clinical applications in cancer and inflammatory disease treatment.

### Progress in the preparation of EV drugs

The preparation process of EV drugs varies according to the source of the EVs. Typically, the preparation of MI-EVs and P-EVs does not involve upstream cell culture. Here, we summarize the progress in EV drug preparation technology based on EVs from cultured cells (Table 5).

**Table 5:** Progress in EV drugs development

Stage	Progress Highlights	Technological advances and considerations	Key challenges
Upstream production	Large-scale culture of EV-producing cells	- Serum-free media reduced contamination and enhanced yields - 3D culture systems (e.g., microcarriers) better mimic in vivo conditions and scale up - cGMP-compliant scalable processes (8–10 L batches) in development	- Limited scale-up beyond pilot scale - Variable EVs yield and quality depending on media and culture system
Downstream purification	EVs separation from complex supernatant	- Differential and density gradient centrifugation - Chromatography (gel filtration, ion exchange, affinity) - Ultrafiltration, size exclusion chromatography - Microfluidics, affinity capture (emerging)	- Low yield and recovery - EVs damage or loss during processing - Purity inconsistency and batch variability
Finished product formulation	Long-term stability of EVs	- Conventional storage in PBS at –80°C - Lyophilization improved room temperature storage - Excipient (e.g., sucrose, trehalose, albumin)-stabilized EVs - New drying methods: continuous, microwave-assisted, thin-film freeze-drying	- EVs degradation over time - Structure disrupted during lyophilization - Lack of mature formulations
Dosage form development	EVs delivery adapted for diverse indications	- Injectable solutions - Gels and aerosols under exploration	- Need for optimal delivery mode per indication

*EV* extracellular vesicle, *EVs* extracellular vesicles

#### Progress in upstream preparation processes

In the upstream production of EVs from cultured cells, culture supernatants containing EVs are collected from cell cultures. The development of large-scale cell preparation technology for EV drugs involves breakthroughs in many technologies, among which the most important are the development of the cell culture medium and the refinement of cell culture methods. The medium composition affects cell growth and significantly affects EV secretion, purity, and recovery. The use of commercial serum-free media can significantly reduce potential EV contamination from serum sources while increasing cell growth and EV production^194^. Traditional two-dimensional cell cultures often have a limited surface area in culture flasks and are difficult to apply to large-scale EV production. Three-dimensional (3D) cell culture, a newly developed culture method, uses 3D structures such as microcarriers to provide surface area for cell attachment and better simulates the growth environment of cells in vivo, improving the viability of large-scale production^195^. At present, the refinement of EV upstream production mainly focuses on the small- and medium-scale-up strategy, and generally, 8–10 L of EVs in solution can be obtained. The development and establishment of cGMP-compliant large-scale manufacturing processes for EV drugs is important for clinical research programs and the future market supply.

#### Progress in the downstream purification process

Downstream purification of EVs refers to removing impurities to isolate the EVs. Although the main components of EVs are lipids, proteins, and nucleic acids, the structure and detailed components of EV subtypes vary greatly. Therefore, EVs obtained by different purification methods have different yields and subtypes, which affects their availability for clinical applications because EV functions are closely related to their subtypes^196^.

Commonly used separation and purification methods for EVs include centrifugation, chromatography, and filtration^197^. Centrifugation methods, such as density gradient centrifugation and differential ultracentrifugation, are based on differences in the density of EVs. By optimizing the centrifugation conditions, EV yields can be effectively improved; however, centrifuging for too long or at a speed that is too high can destroy the EV structure^198^. Chromatography techniques, such as gel filtration, ion exchange chromatography, affinity chromatography, and molecular sieve chromatography, can effectively remove impurities, such as residual proteins and nucleic acids, from EVs. The selection of the flow rate and medium greatly affects the purification. Ultrafiltration, size exclusion chromatography, and other newer technologies are being introduced for use in EV purification. These methods have the advantages of simple operation and low equipment costs while preserving the EV structure and functional integrity^199^. Optimized tangential flow filtration (TFF) protocols—including shear rate control ( < 1,000 s⁻¹), 300-kDa polyethersulfone membranes, and trehalose stabilization—have been reported to increase EV throughput while minimizing aggregation, a critical advancement for clinical-scale manufacturing^200–203^.

The inefficiency of large-scale EV isolation methods is a major obstacle to EV drug development. In commercial production, multiple methods are typically combined to obtain high-quality EVs, but it is still difficult to resolve all the challenges of a specific method, including low separation efficiency, sample loss, low EV recovery and purity, and batch-to-batch variation. Therefore, various novel EV purification technologies have emerged, including affinity capture-based purification methods and microfluidic technology-based purification platforms, which provide better options than current technologies for the downstream purification of EV drugs^204–206^. Microfluidic innovations—from acoustic sorting to immunoaffinity nanoarrays—now enable high-resolution EV isolation with single-vesicle precision, overcoming the throughput–resolution trade-offs of traditional methods^207,208^. These advances are critical for clinical applications requiring rapid, label-free EV purification.

#### Progress in the finished product preparation process

The long-term storage of EVs without loss of function is important for the use of EV products in disease treatment^37^. Studies have shown that the size of EVs decreases when they are stored at 4°C or 37°C for 25 days and that the structure of EVs may change or even degrade^209^. Currently, the traditional method of preserving EVs is storage at –80°C after the suspension of the EVs in saline or PBS buffer^210^. EVs can maintain a stable structure and function when stored in a frozen liquid solution for a short time; however, with time, the EV lipid membrane is gradually disrupted, and the content of the active components gradually decreases as they leak from the EV, resulting in reduced overall biological activity; thus, it is difficult to use EVs in experimental research and clinical applications^211^. Therefore, a more stable and effective preservation method is urgently needed.

Freeze-drying may be a better method for preserving EVs. Studies have shown that after lyophilization, EVs retain their anti-inflammatory and other functional biological activities. Lyophilization also allows for room-temperature storage of EV products, which can greatly improve the accessibility of EV therapy^212,213^. However, during freeze-drying, EVs can be disrupted or denatured, affecting their efficacy^214^. The addition of excipients or optimization of the process can reduce or prevent the adverse effects of freeze-drying on EVs^215^. Freeze-dried EVs containing sucrose and trehalose were shown to maintain complete biological activity, and the addition of human albumin or trehalose during freeze-drying improved the physicochemical stability of EVs stored at room temperature, 4°C, and −80°C^216,217^. Traditional batch-based freeze-drying methods have high time and energy requirements and are not suitable for EVs with complex structures and compositions. Technical methods, such as continuous freeze drying, microwave-assisted freeze drying, and thin-film freeze drying, may improve the speed and efficiency of EV freeze drying^218–220^.

As a relatively new research field, the freeze-drying of EVs has limited reference data, and more research is needed to explore and accumulate related knowledge. In addition, to meet diverse therapeutic needs, EV drugs are being developed using a variety of dosage forms, including aqueous injections, gels, and aerosols, which aim to optimize the delivery mode of EVs on the basis of different treatment scenarios and patient needs, thereby improving the effectiveness and convenience of treatment.

### Challenges in the development of EV drugs

Although there has been significant progress in the technology development and clinical application of EV drugs, they still face a series of challenges. First, the exact mechanism of action at the cellular and molecular levels of natural EVs is difficult to clarify because EV components are complex and rich in many bioactive substances; therefore, it is difficult to completely determine the single or combined active components that provide therapeutic effects against specific diseases. For example, in the treatment of acute lung injury, bone marrow-derived MSC-EVs attenuate lung epithelial cell injury through the MiR-182-5p/MiR-23a-3p/Usp5/Ikbkb axis^221^ and inhibit epithelial cell apoptosis by upregulating SIRT1 expression^222^.

In addition, the technology for GMP-based large-scale production of natural EVs is immature, with critical challenges in terms of cellular sources, scalability, purification efficiency, batch reproducibility, and vesicle heterogeneity. While primary cell cultures face limited passage capabilities in large-scale production, immortalized cell lines or tumor cells are associated with safety concerns^223^. The manufacturing scalability of cell culture and EV purification is challenging, and current industrial-scale yields of EVs rarely exceed 10^13^ particles per liter, which is significantly below therapeutic requirements^224^. The lack of standardized protocols for efficient large-scale EV purification leads to inconsistent particle‒protein ratios (typically ranging from 3×10^8^ to 1×10^10^ particles/μg protein), compromising batch‒to-batch reproducibility^225^. Furthermore, vesicle heterogeneity is present in various vesicle types (e.g., microvesicles vs. exosomes) and particle types (e.g., membrane integrity), which are difficult to distinguish completely during purification^226,227^.

Engineered EVs can somewhat overcome the limitations of natural EVs, such as drug loading and targeted delivery. However, engineering EVs also faces many challenges. The efficacy vs. safety trade-off in EV engineering is a critical consideration for therapeutic applications. While engineering can enhance EV functionality (e.g., drug delivery, targeting, or immunomodulation), it may also introduce risks such as toxicity, immunogenicity, or unintended biodistribution^9,228^. Endogenous loading (via parental cell modification) has relatively stable but low loading efficiency and is associated with a risk of unintended cargo alterations^224,229^. Exogenous loading (postisolation modification) suffers from payload leakage and requires extensive postpurification to remove unencapsulated molecules^230^. Moreover, chemical and physical modification processes can induce structural deformations (15% to 30% vesicle collapse rate) and impair biological activity by denaturing surface proteins^224,229^. The membrane damage caused by electroporation/sonication may lead to EV clearance by the mononuclear phagocyte system, and residual transfection reagents (e.g., lipofectamine) can cause cytotoxicity^231,232^. Quantitative control remains problematic, with typical loading efficiencies ranging from 5% to 30% for small molecules and 0.1% to 5% for nucleic acids^233^. The process for preparing high-purity engineered EVs must be optimized, as engineering modifications may alter the EV membrane topology (e.g., reduced CD63 exposure) and biodistribution patterns^234^. These changes induced by engineered modifications require comprehensive evaluation through advanced characterization platforms combining nanoparticle tracking, proteomic profiling, and functional bioassays. Overall, EV engineering offers immense therapeutic potential, but each modification must be evaluated for dose-dependent toxicity (e.g., cargo leakage), biological compatibility (e.g., immune evasion), and pharmacokinetics (clearance rate, biodistribution).

More importantly, EV drugs still face many challenges in terms of supervision and regulatory science. First, regulatory pathways and drug classifications are complex. Drugs developed on the basis of EVs can be roughly divided into natural and engineered EV drugs. Natural EV drugs with therapeutic functions are treated by the United States Food and Drug Administration (U.S. FDA) and the European Medicines Agency (EMA) as biological products rather than advanced therapeutic medicinal products (ATMPs), whereas the classification of EVs as delivery vehicles depends on the nature of the therapeutic ingredients it loads and delivers (e.g., small molecules, nucleic acids, and antibodies) and may be classified as ATMPs (gene therapies) or biological products^235^.

Second, technical guidelines for EV drug development and evaluation remain inadequate. Research and development of EV drugs is a research hotspot, with many EV drugs currently undergoing clinical trials. However, at present, global health authorities, including the World Health Organization, U.S. FDA, and EMA, have not issued technical guidelines for quality research, quality control, or nonclinical safety evaluation of EVs. Internationally, only group standards exist, such as the ISEV’s MISEV guidelines and country-specific consensus documents^12^. While existing guidelines for cell and gene therapies provide partial frameworks, they inadequately address EV-specific parameters. EV drugs are mixed bioactive molecules wrapped by membranes, which are distinct from cell products and molecular entities at the basic structure level. They are used as both therapeutic entities and delivery platforms, which are typically administered according to different technical guidelines.

Third, tools and methods for evaluating the quality, safety, and efficacy of EV drugs from different sources are urgently needed. Owing to heterogeneity, standardizing EV characterization is challenging. EV drugs prepared from the same raw material are heterogeneous in size, content, and functional characteristics, making evaluating their overall qualities challenging^236^. EV drugs are a mixture of bioactive substances and source-type defined, and unified methods for specific biological activities are lacking and must be designed according to disease type^237^. Furthermore, potency assays for cargo-loaded EVs require multiplexed quantification of both vesicles and payloads^238,239^. Purity and impurities are difficult to define because of the lack of a unified, accurate definition of the active ingredients of EVs and the limitations of existing technologies in distinguishing heterogeneous vehicles (e.g., membrane vs. nonmembrane particles and cargo-loading vs. noncargo-loading particles)^224,240^. In addition, nonclinical research methods, such as pharmacokinetic tracing requiring dual labeling of vesicles and cargos, biodistribution analysis complicated by an endogenous EV background, and immunogenicity assessment requiring species-specific models due to the conserved surface proteins of EVs, present challenges^241,242^.

All these challenges serve as barriers to the clinical translation of EV drugs. Although the existing technical guidelines for cell and gene products can provide a certain guiding framework, there is still an urgent need to develop specific technical guidelines for pharmaceutical research on EV drugs. In the next section, we integrate current knowledge of quality control and nonclinical research on biotechnology drugs, the characteristics of EV drugs, and published technical guidelines for pharmaceutical and nonclinical evaluations of cellular and gene therapy products to propose general principles and key considerations for quality control and nonclinical research on EV drugs from cultured mammalian cells. Research on EV drugs from other sources can also be based on these references.

## Holistic quality control strategy for ev drugs

Quality control is a key component of pharmaceutical research and evaluation of EV drugs and runs throughout the entire drug manufacturing process. The manufacturing process should comply with the basic principles and relevant requirements of the cGMP to ensure that high-quality manufacturing activities are performed in controlled facilities and operated by trained personnel, and all operational steps are recorded in detail to ensure that the manufacturing process continuously meets regulatory requirements^243^. The basic principles of EV drug development can be found in the guidelines for related products, such as cells and gene therapy. However, the physical and chemical characteristics and biological activity of EVs vary widely from those of parent cells, and the production process is very different; therefore, the specific production process and product characteristics of EV drugs should be considered^244^. Generally, the quality control of EV drugs should cover all aspects of production materials, process parameters, in-process controls, release tests, stability studies, process characterization, and validation (Table 6). Only by establishing a holistic quality control system throughout the entire process can the safety and efficacy of EV drug preparations in clinical studies be ensured^111^.

**Table 6:** Overview of holistic quality control strategies for EV drugs

Category	Key Elements	Key Points
Production materials	Cells for production	Refer to Cellular & Gene Therapy Guidances and ICH Q5D
	Raw materials (natural EVs)	Cells, culture media, additives
	Raw materials (engineered EVs)	Plasmids, bacteria, auxiliary cells, viruses, small-molecule drugs, bioactive molecules
	Raw materials, excipients, consumables, packaging materials	Traceable, controllable, and sourced; nonanimal origins recommended; refer to Cellular & Gene Therapy Guidances
Critical process parameters and in-process controls	Key steps in production, IPCs, intermediate testing (if applicable)	Define CPPs and IPCs using quality by design principles to ensure EV consistency and quality; include process performance indexes, microbial safety testing, and other appropriate IPCs
Characterization of EV drugs	Identity	Surface molecular markers (e.g., CD9, CD63, CD81, ALIX), STRs, target molecule identification (for engineered EVs)
	Particle size and concentration	Size and quantity of EV particles
	Morphology and structure	Primary assessment of integrity and purity
	Zeta potential	Surface charge analysis
	Component analysis	Lipids, proteins, nucleic acids (including omics analysis); difficult to exclude interference from coisolates during EV isolation and purification
	Key drug components	Define and detect functional molecules (e.g., miRNA, proteins)
	Purity, contents	Purity may be determined based on membrane integrity and marker positivity; evaluate encapsulation efficiency, loading capacity, and positive rate (for engineered EVs)
	Impurities	Nonmembrane particles, free molecules, nonpositive particles, and drug-free EVs based on product function; Free molecules, nonmembranous particles defined as impurities based on product function
	Biological activity	Based on mechanism of action; evaluated at molecular, cellular, and animal levels; e.g., immunoregulatory, anti-apoptotic, pro-angiogenic (for natural EVs); uptake efficiency, therapeutic molecule function (for engineered EVs)
	General physicochemical properties	pH, osmotic pressure, visible foreign matter, and microbial safety-related quality attributes
Specifications	General considerations	Establish release and stability specifications for drug substance and product (if applicable); test items should generally include identity, particle size distribution and concentration, morphology, biological activity, purity and impurities, microbiological safety attributes, and general attributes; analysis methods should be fully validated
	Acceptable criteria	Determined based on nonclinical and clinical studies; batch release and stability study data combined with statistical methods
	Engineered EV-specific attributes	Encapsulation efficiency, drug load, positive rate
	Reference standards	Encourage reference standards development
Stability studies	Storage, packaging materials, transport	Support shelf-life determination; evaluate stability of intermediates
Process validation	Batch consistency; quality controllability	Validate with ≥3 consecutive batches; usually not validated for virus clearance

*EV* extracellular vesicle, *EVs* extracellular vesicles, *STRs* short tandem repeat sequences, *IPCs* in-process controls, *CPPs* critical process parameters

### Production materials

The production materials used in the EV drug production process include raw materials, excipients, consumables, and packaging materials. As these materials are essential elements of production, their sources and qualities must be traceable and reliable. The materials may carry adventitious agents and introduce or transmit risk; this risk must be strictly controlled, and measures should be taken accordingly. A comprehensive and detailed evaluation and audit of the material suppliers and contract manufacturers must be conducted to ensure that the quality of the materials fully meets the production requirements and to ensure the progress of production activities and the quality and safety of the final products.

The raw materials used for natural EV drug production include mainly cells and production materials such as culture media and various additives. Engineered EV drugs also include components used for endogenous EV modifications, such as plasmids, bacteria, auxiliary cells, and viruses, and for exogenous modifications, such as small-molecule drugs and bioactive molecules.

The requirements for the cell sources used in production and the establishment and qualification of cell banks can refer to relevant technical guidance for stem cell products and ICH Q5D. The use of media and reagents containing animal-derived ingredients in the manufacture of EV drugs introduces potential risks of viral, protein, and foreign particle contamination and immunogenicity^245^. To improve product safety and controllability, materials of nonanimal origin are recommended, such as serum-free media and recombinant growth factors and enzymes, which reduce the risk of contamination and improve the repeatability and standardization of production^194,246^. The composition of the culture medium significantly affects the purity and yield of EVs^247^, and the use of serum-free culture medium can significantly reduce the contamination of EVs by serum sources^248^.

Materials related to engineering modifications require special attention in engineered EV drug production. To obtain or enhance the function or targeting of EV drugs, exogenous or endogenous drug delivery technologies are often used to introduce modified ingredients into natural EV drugs. Plasmids, bacteria, auxiliary cells, viruses, and other raw materials involved in the endogenous delivery process should undergo strict quality control testing to ensure accurate and stable genetic modifications of EV drugs and avoid unnecessary gene mutation or off-target effects^249^. Exogenously delivered chemical small-molecule drugs (e.g., antitumor drugs) and bioactive molecules (e.g., siRNAs, miRNAs, mRNAs, and proteins) should potentially be of pharmaceutical grade, and the introduction of other components should be avoided.

In addition, the requirements for excipients, contact consumables, and medical devices in the EV production process can be found in the Cellular & Gene Therapy Products guidelines.

### Critical process parameters and in-process controls

Critical process parameters (CPPs) are process parameters for which variation impacts critical quality attributes of the product and should be monitored or controlled to ensure that the desired product quality can be achieved. The appropriate in-process controls (IPCs) are key to product quality assurance. Because the composition of EV drugs is complex and product quality is difficult to characterize fully and accurately, it is important to adopt quality-by-design concepts to establish IPCs and CPPs and ensure the consistency of EV yields, quality, and activity^244^. The process parameters may include the cell culture conditions (temperature, dO_2_, pH, and CO_2_), physical parameters and time range of each production step. Intermediate products at the proper process steps should be tested for quality-related parameters, such as cell morphology and viability and EV characteristics, content, and recovery rates, with acceptable ranges set to provide a basis for action limits. Microbial safety attributes, including bioburden, sterility, mycoplasma, and endotoxins, should be tested at appropriate stages of the production process.

### Characterization of EV drugs

#### Physicochemical properties of EV drugs

Natural EVs are mainly prepared from biological fluids (e.g., milk), tissue lysates (e.g., plants), and cell cultures. It is difficult to obtain single-component EVs as final products from these complex raw materials via current technology. In addition, EVs prepared from the same raw materials exhibit great heterogeneity in size, content, and functional characteristics. Therefore, the physicochemical properties of EV drugs should be comprehensively evaluated considering individual EVs, the EV population, and non-EV components.

On the basis of the latest research in the field^12,125^, the main physical and chemical characteristics of natural EV drugs are summarized as follows:

i. Identity: The protein molecules CD9, CD63, CD81, and ALIX are considered markers of EVs and can be detected by western blot and flow cytometry^250^. Furthermore, the biological source of a specific EV can be identified by microsatellite DNA (also called short tandem repeat sequences, or STRs). In addition, component identification of target molecules could be performed on engineered EVs, such as detecting target proteins with specific antibodies and detecting target nucleic acid molecules with specific nucleic acid probes.
ii. Particle size and concentration: The size and quantity of EV particles are the most basic physical attributes of EVs. Nanoparticle tracking analysis (NTA) and nanoflow cytometry (NanoFCM) are the most commonly used methods for measuring the size and number of EV particles in EV samples; these methods also reflect the correlation between the particle size distribution and the particle number^251–253^.
iii. Morphology and structure: Morphology and structure are basic characteristics of EVs as vesicles and important parameters affecting their functions. Microscopic techniques such as scanning electron microscopy, transmission electron microscopy, cryo-electron microscopy, and atomic force microscopy are usually used to analyze the morphology and microstructure of EVs. These techniques can provide high-resolution images of EVs for analyzing their morphology and ultrastructure in detail^254^.
iv. Zeta potential: The EV surface charge is also an important parameter. The surface charge mainly affects the interactions between EVs and other molecules^255^. The zeta potential of EVs is usually measured by laser polygon scattering, microfluidic dynamic light scattering, and electrophoresis-based zeta potential analysis^255,256^.
v. Component analysis: EVs consist of a membrane and its cavity contents, which are composed mainly of biomolecules such as lipids, proteins, and nucleic acids. For a given amount of EVs, the total quantity of lipids, proteins, and nucleic acids can be quantified, and the specific types and abundance of other various components can be qualitatively analyzed via omics technology^257–260^. Notably, it is difficult to exclude interference from coisolates during EV isolation and purification, as the components of coisolates are also proteins, nucleic acids, lipids, and other biomolecules.
vi. Key drug components: Molecules known to play key therapeutic roles in EV drugs can undergo quantitative detection, such as the use of quantitative polymerase chain reactions to detect miRNAs, mRNAs, and other nucleic acid molecules and western blotting and enzyme-linked immunosorbent assays to identify specific proteins.

#### Purity, contents and impurities

EVs are a complex of multiple active components, and their membrane integrity is required for their biological function. Owing to the limitations of production processes, the final EV products usually contain EV-coated particles, membrane-free particles, and free molecules. Therefore, the purity and content of EV drugs can be characterized via the use of EV particles with intact membranes. Currently, the purity and content of EV drugs can be quantified via the percentage and number of particles with intact membranes, respectively. The particle size and concentration determined by NTA or NanoFCM cannot distinguish other particles from EVs with similar membrane integrity, and electron microscopy analysis cannot be used to quantify the number of EVs with intact membranes. Therefore, the analysis of membranous particle content in EV samples is usually based on quantitative fluorescence detection techniques of enzyme activity^261^. EV particles with intact membranes may not be completely accurate because of the presence of particles with disrupted or destroyed membranes, which are difficult to distinguish from EV particles with intact membranes in practical testing.

The EV membrane protein markers CD9, CD63, and CD81, such as the number and proportion of CD9, CD63, and CD81 single-positive, double-positive, and triple-positive particles, can be used to evaluate EV purity and content via flow cytometry^254^. The evaluation based on positive markers should clarify whether it is based on detecting particles with membranes. The positive marker determination of particles with membranes will be important for accurately quantifying EV drug purity and content in the future, but related technologies need to be further developed and optimized.

The quantitative evaluation of engineered EV drugs can also be performed from the perspectives of encapsulation efficiency, loading capacity, and positive rate^262^. The encapsulation efficiency refers to the ratio of the amount of drug successfully loaded into the EV to the amount of drug in the preparation system. The encapsulation efficiency is used to evaluate the efficiency of drug loading and is applicable mainly for evaluating exogenous drug loading. The loading capacity refers to the quantity of drug loaded per unit of EV. Theoretically, a greater loading capacity is associated with a lower amount of EVs required for administration. The units for loading capacity vary according to the drug being loaded, such as the mole number, mass, and copy number. The positive rate refers to the ratio of EVs successfully loaded with drugs to total EVs and is also used to evaluate the drug-loading efficiency of EVs. The loading capacity and positive rate can be used for endogenous and exogenous drug loading evaluations. At present, technology for quantitatively evaluating engineering EV loading is in its infancy and is a focus of future research.

In natural EVs, membranous particles without membrane integrity, nonmembrane particles, and free molecules (nucleic acids, proteins, and lipids) can be defined as impurities in a purity analysis on the basis of membranous particles with intact membranes. Nonpositive particles can be further defined as impurities via particle purity analysis on the basis of positive markers. When EVs are only used as drug delivery vehicles, drug-free EVs are also considered impurities, and when EVs are used as therapeutic drugs and delivery vehicles, drug-free EVs are not controlled as impurities. At present, there is no reliable method for determining the impurity content of EV drugs. In general, an impurity analysis of EV drugs depends on an accurate definition of the active ingredients of the EVs, and their control depends on technological improvements in the manufacturing process.

#### Biological activity

Biological activity is the key quality control attribute for evaluating the clinical effectiveness of EV drugs, and it is also a reflection of their highly ordered structures. The biological action of EV drugs may involve multiple targets that affect multiple pathways, with different types of EV products producing different biological functions. A robust, sensitive, and dose‒responsive evaluation method that reflects the clinical mechanism of action should be established. Products with multiple active components should be studied separately to establish methods for determining biological activity that reflect different mechanisms of action of each component.

The biological effects of EVs include immune regulation, angiogenesis, cell proliferation, cell migration, and anti-apoptosis regulation. When evaluating the biological activity of EV drugs, the most important task is establishing relevant detection methods at the molecular, cellular, and organism levels on the basis of the mechanism of action and product characteristics. For example, if EV drugs are developed from MSC-EVs, which are thought to improve the survival rate of sepsis patients through anti-inflammatory and tissue repair mechanisms, then dose-dependent effects of EVs on the survival rate of sepsis model animals, inhibitory effects on inflammatory macrophages and apoptosis, and proliferation-promoting effects on other tissue cells can be observed. Moreover, the effects of EVs on the expression of key molecules in inflammatory and proliferation-related pathways can be analyzed at the molecular level.

The biological activity of engineered EVs can also be evaluated by designing specific biofunctional experiments on the basis of the properties of the specific drugs. EV-loaded drugs are mainly therapeutically active molecules or tissue- or cell-targeting molecules. In quality studies of engineered EVs, the dose‒dependent response of therapeutically active molecules in cells or animals should be evaluated. EVs engineered with tissue- or cell-targeting molecules should be evaluated in both cell and animal models to determine the efficiency of their uptake by target tissues and cells. The uptake efficiency of engineered EVs loaded with therapeutically active molecules and tissue- or cell-targeting molecules by target cells should be evaluated, and their effects on cell behavior should be evaluated at the same time.

In addition to these quality studies, EV drug analyses should also include general physicochemical properties, such as pH, osmolality, and visible particles, and microbial safety-related quality attributes, such as the presence of bacteria, fungi, endotoxins, mycoplasma, or viruses.

### Specifications establishment

Specifications, as an important part of product quality control, are generally determined on the basis of product characteristics and quality studies. In view of the complex structure and special quality attributes of EV products, specifications should be comprehensively established through risk assessment on the basis of development experience, quality studies, understanding of the critical quality attributes of the final products, and the continuous accumulation of knowledge of the products in combination with nonclinical and clinical data. The core principle of quality specifications is to ensure, through adequate testing, that the product meets the specified requirements in terms of identity, purity, safety, and efficacy^263^. If the production of drugs is divided into two stages, drug substances and drug products, then release and stability specifications should be established separately. The analysis methods should be fully developed and validated, particularly for newly developed methods. If comprehensive quality control testing cannot be completed owing to the short shelf life of the product, simplified release testing procedures can be adopted instead. In these cases, the rationale for simplifying procedures must be clearly justified, and the release information must be supplemented with appropriate process monitoring and extensive process validation, which may include the collection and analysis of real-time process control data to ensure performance within critical quality attribute ranges.

Testing items to evaluate EV quality should be based on product quality studies and a full understanding of the production processes and clinical indications while taking into consideration the product characteristics and the current scientific understanding and consensus. As the study continues through drug development (e.g., from the preclinical stage to the clinical stage), process-related information should be gradually accumulated, and the test methods should be gradually improved to support the quality control requirements of each stage. The product quality control requirements of confirmatory clinical trials should be consistent with those of commercial production. EV specifications should generally cover EV identity; particle size distribution, concentration, and morphology; biological activity; purity and impurities; microbiological safety attributes (sterility, endotoxins); and general tests (e.g., zeta potential, pH, osmolality, visible particles, fill volume). Engineered EV drug specifications should also include encapsulation efficiency, drug loading, positive rate, and drug loading distribution. Special dosage forms require additional tests consistent with the characteristics of the dosage form. Specific test items should also be determined on the basis of the final product type, production process, stability studies, and risk assessment.

Reference standards (RSs) are important for ensuring the consistency and accuracy of test results. RSs may be used in analytical methods, and the development of RSs for product quality control is encouraged. The RSs used for analysis should be representative, traceable, fully characterized via validated analytical test methods, and qualified.

### Stability studies

Stability studies are important throughout drug research and development and support drug marketing and postmarketing studies. These studies constitute the basis for establishing the shelf-life of products. Stability studies can be used to justify production process, formulation, and packaging material decisions, and they are also the basis for product specifications. The basic principles of EV drug stability studies should refer to cell therapy product requirements^264^. The study protocol should be designed according to the characteristics of EVs, clinical application requirements, and specific storage, packaging, and transportation conditions to ensure their stability and safety throughout the supply chain^265^. In addition, physical, chemical, and microbial stability studies should be conducted on intermediate products that are temporarily stored during the production process to verify their appropriate storage conditions and duration. These studies help clarify how EVs behave in different environments and provide critical data for optimizing production and storage processes^217^.

### Process validation

During the entire process of EV drug production, comprehensive process validation is required via multiple consecutive batches (at least three batches) to assess the repeatability and consistency of the process. For process validation batches, intermediate and finished products should be tested, analyzed, and characterized more extensively than routine batches to provide a basis for establishing process controls and release criteria for routine batches. Because of the size and composition (lipid and protein) of EVs, existing virus clearance technologies, such as chromatography, nanofiltration removal, and detergent inactivation, may not be suitable for EV drugs. Therefore, EV drugs are usually not validated for virus clearance. However, because EV products lack a terminal sterilization process, strengthening microbial safety risk controls in raw materials and production processes is essential.

## Key aspects of nonclinical studies of ev drugs

**Fig. 4:**
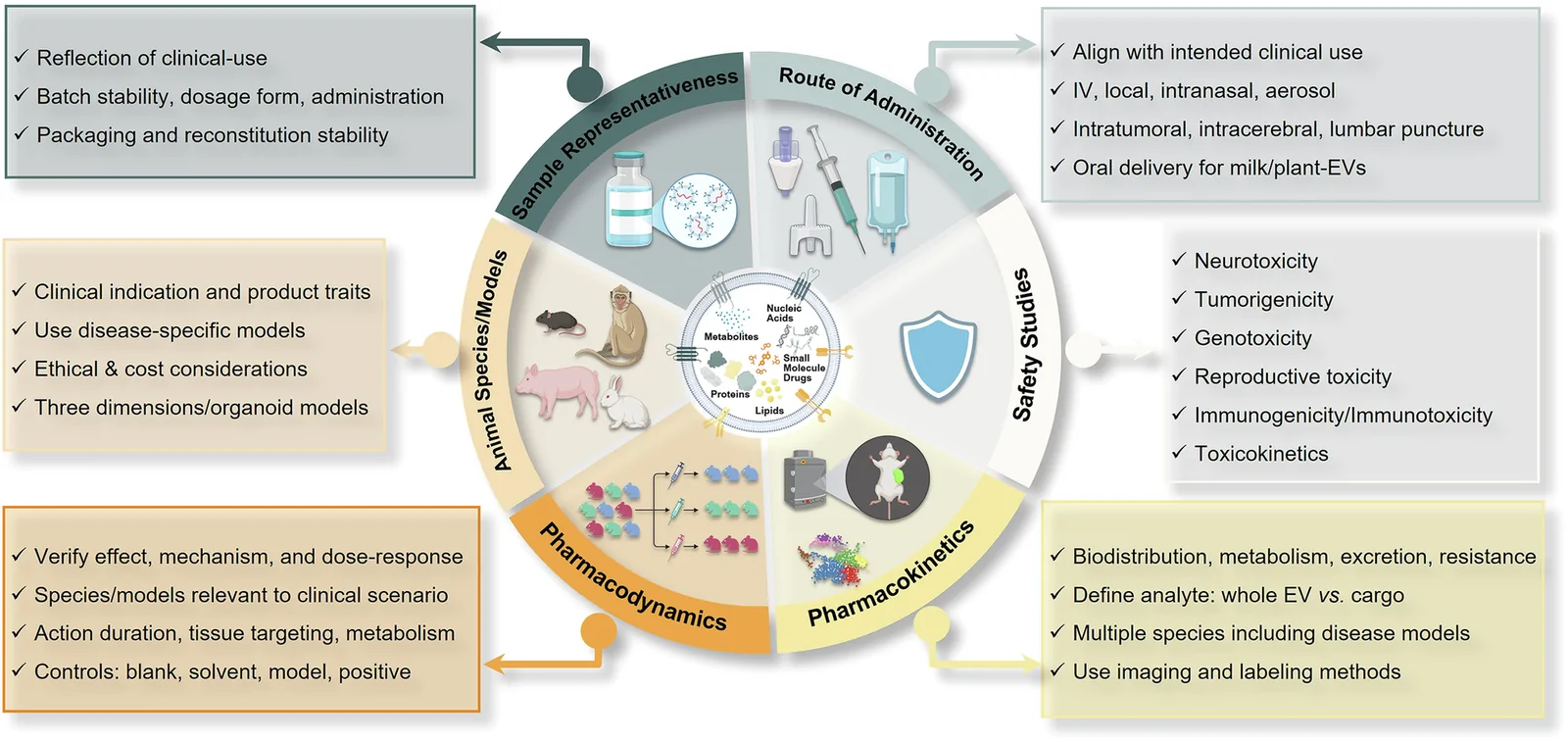
Key aspects of nonclinical studies of extracellular vesicle (EV) drugs. The key concerns regarding the representativeness of samples, animal species/models, and routes of administration are introduced for developing a nonclinical study strategy for EV-based drugs. The critical aspects of pharmacokinetics, pharmacodynamics, and safety evaluations in nonclinical assessments of EV drugs are outlined. The representative schematics in this figure were created with BioRender.com

Nonclinical studies of EV drugs are important for evaluating their safety and effectiveness as new drugs and usually include pharmacodynamics, pharmacokinetics, safety evaluations, and other studies^266^. For nonclinical studies of EV drugs, risk-benefit-based analysis strategies should be followed, combined with the principles of specific analysis on the basis of specific problems and testing all that should be tested. Currently, drug regulatory agencies in China and internationally have not yet issued relevant guidelines for nonclinical studies on EV drugs. Here, we discuss key issues in nonclinical studies of EV drugs (Fig. 4).

### Key points of concern for developing a nonclinical study strategy

#### Representativeness of samples

The test article used in nonclinical studies should be representative of the sample intended for clinical use. The manufacturing process of EV drugs, the stability of samples in different batches, and the characteristics of the product itself may affect the design, results, and conclusions of nonclinical studies. Therefore, the samples should be fully representative. In nonclinical studies, in consideration of specific administration scenarios corresponding to specific indications, the quality, dosage form, administration conditions, and route of administration of the test article should be fully assessed to represent the sample under study. For example, in nonclinical studies, the possible impact of product packaging materials on EV aggregation must be considered. In addition, when EV drug preparations are frozen or lyophilized, the in-use stability of the preparation after reconstitution should also be considered. If the subsequent preparation process changes, the similarities and differences between the nonclinical study samples and the samples intended for clinical trials should be determined, including their possible impact on human efficacy and safety.

#### Animal species and models

The selection of animal models is a key step in determining the success or failure of nonclinical studies. The selection of animal models is mainly based on product characteristics, clinical application scenarios, and the correlation between the pathophysiological status of the animal model and clinical indications. There are significant differences in physiological metabolism, the immune response, and other factors among different species^267,268^. Therefore, appropriate species selection is of vital importance.

The animal species and model selection for pharmacodynamic studies should be consistent with the disease application of EV drugs. In cancer research, mouse-based xenograft tumor models and genetically modified tumor models are widely used to evaluate the antitumor effects of EV drugs^269^. In neurodegenerative disease studies, disease-specific genetically modified mice are commonly used to study the neuroprotective effects of EV drugs and the ability of EVs to cross the blood‒brain barrier^270^. Cardiovascular disease research often uses pig and rabbit models, such as in the evaluation of myocardial repair by EV drugs, because their cardiovascular systems are more similar to those of humans than are those of other animals^271^. Studies on EV drugs for immune-related diseases usually use animals with intact immune functions, such as rat or rabbit models of rheumatoid arthritis or diabetes^272^. Furthermore, animal species selection should consider the route of administration of a specific EV drug. The tissue distribution of EV drugs varies in different animals. Different modes of administration may need to be verified by using different species of animal models to ensure that the distribution, absorption, and efficacy of EV drugs more accurately reflect these factors in humans^273,274^.

Rationality and applicability are the primary parameters for selecting animal species and models for safety evaluations of EV drugs. Ethical and economic factors are also important considerations. Rodents (e.g., mice and rats) reproduce quickly and are inexpensive; therefore, they are often used for preliminary pharmacological and toxicological studies. However, their immune systems and metabolic rates differ significantly from those of humans^275^. The physiological structure and immune response of nonhuman primates are highly similar to those of humans, and they provide unique advantages in preclinical studies of EV drugs, but they are costly and accompanied by ethical issues^276,277^. Alternative models, such as 3D cell culture and organoid technology, which can simulate the human tissue environment to a certain extent, have become important tools in nonclinical research on EV drugs^278^.

#### Route of administration

The route of administration of EV drugs is a crucial factor in nonclinical studies, and its determination is directly related to the therapeutic effect and feasibility of future clinical applications^279^. The administration mode must be selected by considering the disease type, location of the disease, and in vivo distribution of EV drugs under different routes of administration. The selected route of administration should be as consistent as possible with the expected clinical use^280^. Common modes of EV drug administration include intravenous, local, intratracheal or aerosol, intranasal or nasal, and oral administration.

Intravenous administration is the most common means of administering EV drugs and allows rapid distribution throughout the body via blood circulation. Intravenous administration is suitable for diseases related to organs with an abundant blood supply, such as the liver, spleen, and lung, and for systemic diseases, such as immune system disorders and tumors. Local injections are suitable for local diseases, such as subcutaneous injection for skin diseases, intralymph node injection for lymph node-related diseases, and intra-articular injection for joint diseases. Local injections allow EV drugs to act directly on the lesion site, thereby improving the local treatment effect. Intratracheal or nebulized administration can be used to treat lung and respiratory diseases. EV drugs administered intratracheally can act directly on the lungs and respiratory tract, reducing off-target effects on other organs. Intranasal administration is primarily used to treat central nervous system disorders, which require drugs to cross the blood‒brain barrier. When administered intranasally, EV drugs are absorbed through the nasal mucosa and enter the bloodstream or act directly on the central nervous system. Oral administration provides high potential for long-term and convenient treatment. However, EV drugs derived from cultured cells may be susceptible to digestive enzymes in the gastrointestinal tract; milk and plant-based EV drugs have greater application potential in this regard^281^.

In addition to these common dosing modalities, several other EV drug delivery modalities target specific disease sites^282^. Lumbar puncture, intracranial injection, and intracerebral catheterization can deliver EV drugs directly to the central nervous system, bypassing the physiological barrier of the blood‒brain barrier and thus improving the effectiveness of the drugs^283^. In tumor therapy, intratumoral injection can achieve precise targeted delivery of EV drugs at tumor sites, thus increasing the local drug concentration and reducing systemic exposure and side effects^284^.

### Pharmacodynamics studies

Pharmacodynamic studies should use reliable methods to verify the physiological effects, mechanism of action, and dose effects of EV drugs. Biological activity evaluation methods should be established on the basis of the characteristics of specific EV drugs, which can then be used as indicators to evaluate their efficacy in specific clinical indications. The study design should fully consider factors such as the mechanism of action, type of indication, length of the disease cycle, and mode of administration of EV drugs. The characteristics, targeting, and metabolic half-life of the EV drug should be considered in pharmacodynamic studies to select in vitro and in vivo models that strongly correlate with specific clinical indications^285^. Efficacy studies of EV drugs should focus on the following points:

*i* Selection of animal species: The selection of animal species and models should be based on scientific and reasonable evidence. The characteristics of EV drugs and their clinical application should be fully considered when selecting relevant animals for nonclinical in vivo studies.

*ii* Design of the dosing regimen: The most important purpose of a pharmacodynamic study is to provide a data basis for the appropriate administration route, administration procedure, and dosage of a clinical trial. Therefore, in nonclinical efficacy studies, it is necessary to consider the commonalities and characteristics of the specific EV product, fully consider the relationship between the administration route and efficacy and the relationship between the administration frequency, time window, and efficacy, and then determine the optimal dosing regimen. For example, EV drugs generally exhibit “short time–high efficiency” in vivo. Moreover, because the retention time of an EV drug in tissue, organs, or target organs is longer than the time at which the drug circulates in the blood, the dosing frequency cannot be determined simply by the plasma clearance half-life^286^. Therefore, the dosing frequency and dosage must be appropriately adjusted according to the metabolic rate of the drug and the characteristics of the indication.

*iii*Pharmacodynamic evaluation on the basis of the mechanism of action of EVs: According to the classification of different EV drugs, the focus of pharmacodynamic studies may vary slightly. For products in which EV itself is the drug, the active molecule of the drug is generally a combination of multiple components, and its mechanism of action cannot be verified through a single target. A comprehensive biological function analysis is needed, and reasonable pharmacodynamic evaluation indicators should be set to evaluate the overall efficacy of EVs. When the target molecule is loaded with engineered agents, the functional pharmacodynamic effect produced by the target molecule should be evaluated against that of nonengineered EV drugs. For engineered EV drugs that use EVs as delivery vehicles, the focus should be on the functional potency of the loaded molecule. A control group should be established for pharmacodynamic studies. A negative control (blank control), solvent control, model control, and positive control should be established according to the specific EV drug type and requirements of the protocol.

### Pharmacokinetic study

Exploring the pharmacokinetic behavior of an EV drug, including its biodistribution, clearance rates, and metabolic pathways in vivo and determining its half-life, is critical for evaluating its safety and efficacy^287^. The following key points should be considered in the pharmacokinetic study of EV drugs:

*i* Clarification of the object of the study: When EVs themselves are used as drugs, the entire EV can be regarded as active ingredients; if the EVs are used as delivery vehicles, the contents they carry can be used as the active ingredients. A comprehensive study on EVs and the drugs they carry requires attention to the behavior of both the EVs in vivo and the release, metabolic pathway, and excretion mechanisms of the drugs they carry^288^. Analytical subjects should be selected according to different research objectives. If a study focuses on the distribution, half-life, and persistence of EVs, dynamic changes in EVs should be monitored during the analysis; if the study focuses on the release rate, bioavailability, and efficacy of the drug component in EVs, the drug component should be the primary object of analysis^289^.

*ii* EV drug absorption and distribution studies: Single-dose (including multiple doses if necessary) pharmacokinetic and tissue distribution tests should be considered in healthy animals of various species related to the indications. Because the pharmacokinetics of EV drugs may vary among different species, multispecies animal studies may be conducted to help predict or evaluate toxicity test dose‒response relationships. Furthermore, some EVs exhibit a “homing” effect in disease models^290,291^, which results in different pharmacokinetic behaviors than those in normal animals. Pharmacokinetic and tissue distribution tests involving the administration of single doses (and multiple doses if necessary) should be considered in disease model animals.

*iii* EV drug metabolism, excretion, and release studies: Due to the technical limitations of current pharmacokinetic methods, it is still quite difficult to comprehensively determine the metabolism, excretion, release, and pharmacokinetic parameters of EV drugs through existing technologies^285^. EV drugs may involve EVs themselves and their key active pharmaceutical ingredients. When these studies are conducted, the in vivo distribution and pharmacokinetic parameters of the EV itself and the drug may need to be distinguished^292^. The payloads of engineered EV drugs that use EVs as delivery vehicles and contain accurately measurable payloads (e.g., synthetic polypeptides, small-molecule drugs, antisense oligonucleotides) should be evaluated for metabolic, excretion, release, and pharmacokinetic parameters. These aspects of the study can refer to specific molecular drug study models. The pharmacokinetic parameters of the active ingredients must be determined, but whether the pharmacokinetic parameters of EVs need to be determined separately is open to discussion. Compared with chemical drugs, monoclonal antibodies, and cellular products, pharmacokinetic studies based on EV drugs are still in the initial stage of development and face many challenges. This requires specific analysis to be performed on the basis of the specific EV products; these analyses may have difficulties related to immature methods for labeling, tracing, and detecting EVs. Currently, EVs are typically labeled with radioisotopes, fluorescence, or nanoparticles, and their distribution and persistence in vivo can be monitored by magnetic resonance imaging, fluorescence in vivo imaging, and flow cytometry^293,294^. However, these methods are technically limited and cannot accurately and quantitatively characterize the results. Therefore, accurate pharmacokinetic studies of the drug metabolism of EVs urgently require tremendous effort from researchers.

### Safety studies

The general principles for safety evaluations of EV drugs are based on the technical guidelines for the Research and Evaluation of Cell and Gene Therapy Products and comply with Good Laboratory Practice (GLP) guidelines. Studies and tests conducted under non-GLP conditions should be described and evaluated for the reliability, integrity, and impact of the test results on the overall safety evaluation of the drug. Both general toxicity tests and personalized safety evaluation systems should be established on the basis of different EV sources. The following provides references for key concerns in EV safety studies, including neurotoxicity, genotoxicity, immunogenicity, immunotoxicity, and tumorigenicity.

#### Neurotoxicity

EVs can cross the blood‒brain barrier and affect the central nervous system. For example, MSC-EVs derived from MSCs provide neuroprotection by directly exerting anti-inflammatory and antioxidation effects in neurodegenerative disease models, such as Parkinson’s disease and Alzheimer’s disease models^295^. EVs derived from tumor cells may cause neuroinflammation and nerve injury in animal models by transporting proinflammatory molecules (e.g., IL-6 and TNF-α)^296^. However, few systematic studies have evaluated the neurotoxicity of EV drugs, and the long-term effects of these drugs on the nervous system require further evaluation. To perform neurotoxicity studies of EV drugs, direct investigations can be undertaken on specific models and combined with other toxicity studies that focus on neurotoxicity.

#### Tumorigenicity and oncogenicity

Unlike cell therapy products, EVs are not living organisms and are therefore not tumorigenic. However, because EVs are rich in bioactive molecules, including noncoding RNAs with transformation potential, tumorigenicity should be evaluated. Short- and medium-term animal studies have indicated a low risk of tumorigenicity^297,298^, but the risk of tumorigenicity associated with repeated long-term dosing remains unclear. Therefore, during long-term toxicity testing, tumorigenicity should also be monitored to fully evaluate the implications of EV drugs. Because EVs can promote cell proliferation and angiogenesis and prevent apoptosis by transferring endogenous bioactive components, especially tumor cell-derived EVs^299^, the risk of promoting tumor progression in specific patients (e.g., patients with cancer) given their specific product characteristics should be considered. If necessary, tumor-promoting tests should be conducted.

#### Genotoxicity

EVs are not directly integrated into the host genome and therefore theoretically have a low risk of genotoxicity. If the loaded compound in engineered EVs is a completely new compound, genetic toxicity tests must be conducted. EV drugs are classified as gene therapy drugs, and special attention should be given to genetic toxicity studies. Although studies have shown that stem cell-derived EVs have negative genotoxicity test results, more comprehensive genomic safety monitoring is recommended^300^. Therefore, genotoxicity evaluations are currently recommended for all types of EV drugs.

#### Reproductive toxicity

Only a few studies have evaluated the reproductive toxicity of EVs. Reproductive toxicity testing assesses the fertility, embryonic development, and health of offspring. The available reproductive toxicity data on EVs indicate that they do not exhibit obvious reproductive toxicity and may have protective effects on the reproductive system^301^. For example, MSC-EVs were shown to improve the status of premature ovarian failure in rats^302^. However, there is insufficient research to determine whether EVs cause long-term adverse effects on reproductive function, and more cross-species, long-term safety experiments are still needed. In general, nonclinical evaluations of reproductive toxicity are not necessary for EVs themselves and can be performed during clinical trials in accordance with relevant guidelines (e.g., ICH M3 [R2]). For EV drugs classified as gene therapy drugs, the Technical Guidelines for Nonclinical Research and Evaluation of Gene Therapy Products can be referenced.

#### Immunotoxicity and immunogenicity

Immunomodulation is one of the main goals of stem cell-, milk-, and plant-derived EV treatment and is the basis of treatment for inflammation-related diseases. Therefore, EVs theoretically impact the immune system. Current studies have not indicated any immune-related safety risks for EVs^303^. However, immune-related assessments remain essential, and long-term immunotoxicity findings must be confirmed to determine their safety in different therapeutic settings. Nonclinical safety evaluations should focus on potential immune-related safety risks associated with engineered active ingredients. The immunogenicity of EVs is closely related to their origin and surface modifications. Current studies have shown that MSC-EVs have low immunogenicity and that MI-EVs and P-EVs have even lower immunogenicity. However, EV drugs must still be evaluated for immunogenicity. By analyzing the immunogenic components of EV surface proteins, the possible immune responses they may trigger in vivo are more likely to be predicted. In addition to immunogenicity concerns, studies have indicated that allogeneic antigen-presenting cell-derived EVs can bind T-cell receptors, activate T cells in vivo and sensitize mice to alloantigens under inflammatory conditions^304^. Therefore, the antigenicity of EV-based therapeutics should be carefully evaluated, particularly for EVs derived from immune cells or those engineered to carry antigens^304–307^.

#### Toxicokinetics

It is currently impossible to conduct comprehensive toxicokinetic analyses of EV drugs because of the limits of analytical technology, detection sensitivity, and specificity. These evaluations should be conducted within the framework of a risk-based analysis, and if no risk per se is considered relevant for the EV drugs, concomitant plasma concentration data from repeat-dose toxicity studies of the EV drugs should be collected.

In general, the ability to perform nonclinical safety assessments of EV drugs is gradually improving, with studies demonstrating that EVs have a low safety risk for neurotoxicity, tumorigenicity, genotoxicity, reproductive toxicity, immunotoxicity, and immunogenicity, particularly stem cell-derived EVs^308^. However, further long-term and cross-species studies are needed to fully assess the safety of EVs in different treatment scenarios. Future studies should focus more on elucidating the toxicity mechanisms of EVs and long-term safety monitoring to ensure the safety of their clinical use.

## Conclusions

As one of the main research hotspots, EV drugs have strong application potential for treating multiple diseases, including respiratory and nervous system diseases, severe acute inflammation, and tumors. EV drug research and development are rapidly advancing. More basic research is being conducted to analyze the biological functions and mechanisms of action of EVs in depth. Efficient engineering technology for improving the targeted delivery accuracy and therapeutic effects of EVs continues to be developed. Industrial transformation is focused on optimizing EV production, isolation, purification, formulation preparation, and quality control to ensure the safety and effectiveness of EV products produced at a large scale. However, the successful clinical translation of EV drugs still faces numerous technical and regulatory challenges, which restrict the R&D process to varying degrees. Drug regulatory science is a “bridge” to promote the translation of innovative drugs from basic research to industry. Without advances in regulatory science, promising drugs may be abandoned in the development process; conversely, substantial money and time can be wasted when evaluating a product that later proves unsafe or ineffective. The technical guidelines for drug R&D form an important part of regulatory scientific research. However, owing to their wide range of sources, complex components, and diverse types of EV drugs, global drug regulatory authorities have not issued guidelines for pharmaceutical and nonclinical research on EV drugs. In view of this situation and the urgent needs of institutions performing R&D on EVs, we reviewed the latest research and development progress of EV drugs and proposed the general principles and key considerations of quality control strategies and nonclinical evaluations, with the expectation of providing valuable references for the development and evaluation of EVs and related products.
