# Correction: Extracellular vesicle-based drug overview: research landscape, quality control and nonclinical evaluation strategies

**DOI:** 10.1038/s41392-026-02999-5

**Published:** 2026-09-21

**Authors:** Gangling Xu, Jing Jin, Zhihao Fu, Guangming Wang, Xinhua Lei, Jun Xu, Junzhi Wang

**Affiliations:** 1 National Institutes for Food and Drug Control, Beijing, China; 2 State Key Laboratory of Drug Regulatory Science, Beijing, China; 3 National-Local Joint Engineering Laboratory of Animal Models for Human Disease, The First Hospital of Jilin University, Jilin, China; 4 East Hospital, Stem Cell Research Center, School of Medicine, Tongji University, Shanghai, China

**Keywords:** Drug regulation, Mesenchymal stem cells

Correction to: *Signal Transduction and Targeted Therapy* https://doi.org/10.1038/s41392-025-02312-w, published online 14 August 2025

After publication of the review article,^1^ the authors identified three descriptive errors related to the particle sizes of microvesicles and exosomes upon thorough re-examination of the published manuscript. These discrepancies arose from unintentional clerical mistakes introduced during the manuscript drafting phase.

There are three places to be corrected. Two are in the second paragraph of the Introduction. The sentence “Microvesicles are formed and released after intracellular substances are directly enveloped by the cell membrane, with sizes ranging from 30 to 150 nm in diameter. Exosomes are formed by endocytosis, in which substances are enveloped by the plasma membrane to form membranous particles inside an endosome. The endosome fuses with the cell membrane and releases membrane-bound particles, which range in size from 100 to 1000 nm^13^.” should be corrected as “Microvesicles are formed and released after intracellular substances are directly enveloped by the cell membrane, with sizes ranging from 50 to 1000 nm in diameter. Exosomes are formed by endocytosis, in which substances are enveloped by the plasma membrane to form membranous particles inside an endosome. The endosome fuses with the cell membrane and releases membrane-bound particles, which range in size from 40 to 160 nm^13^.”; Another one is in Table 1, row “Size distribution”, which should be “40–1000 nm; overlap between exosomes and microvesicles” rather than “30–1000 nm; overlap between exosomes and microvesicles”.

**Table 1:** The heterogeneity of EVs (microvesicles and exosomes) parameters

Heterogenous parameter	Specific features	Influencing factors	Implications for downstream development
Size distribution	40–1000 nm; overlap between exosomes and microvesicles	Biogenesis pathway, isolation methods	Affects separation strategies and purity assessment
Surface markers	Shared expression of CD9, CD63, CD81 across subtypes	Cell type, phenotypic changes	Impacts identification and subpopulation profiling
Cargo composition	Uneven distribution of proteins, RNA, DNA, and lipids	Parental cell state, environmental conditions	Determines functional effects and therapeutic indications
Biological function	e.g., Pro- or anti-inflammatory effects, immune modulation, metastasis	Cargo content, recipient cell interaction	Guides clinical use and therapeutic design
Method sensitivity	Varying detection or capture efficiencies across isolation and analysis tools	Instrument sensitivity, parameter selection	Contributes to interlab variability and reproducibility issues

*EVs* extracellular vesicles

The correct size specifications for microvesicles and exosomes have been prepared to amend the erroneous descriptions accordingly, and the original article has been updated with the revised content. The core conclusions presented in this review remain entirely unaffected by these minor adjustments.
